# Disposal practices of cigarettes and electronic nicotine products among adults, findings from Wave 6 (2021) of the PATH Study

**DOI:** 10.1371/journal.pone.0338007

**Published:** 2025-12-09

**Authors:** Eva Sharma, Johanna Dubsky, Katherine V. Garcia-Rosales, Michael Halenar, Adam F. Benson, Alexander Maki, Andrea L. Ruybal, Aura Lee Morse, Michael D. Sawdey, Rudaina Alrefai-Kirkpatrick, Silvana Skara, Susana Addo Ntim, Hoshing W. Chang, Ther W. Aung, Heather L. Kimmel, Jennifer L. Pearson, Maansi Bansal-Travers, Kristie A. Taylor, Andrew Hyland, Thomas E. Novotny

**Affiliations:** 1 Westat, Rockville, Maryland, United States of America; 2 Center for Tobacco Products, United States Food and Drug Administration, Silver Spring, Maryland, United States of America; 3 The MetroHealth System, Cleveland, Ohio, United States of America; 4 National Institute on Drug Abuse, National Institutes of Health, Bethesda, Maryland, United States of America; 5 Department of Health Behavior, Policy, and Administration Sciences, School of Public Health, University of Nevada -- Reno, Reno, Nevada, United States of America; 6 Department of Health Behavior, Division of Cancer Prevention & Population Sciences, Roswell Park Comprehensive Cancer Center, Buffalo, New York, United States of America; 7 School of Public Health, San Diego State University, San Diego, California, United States of America; Federal University Otuoke, NIGERIA

## Abstract

**Background:**

Tobacco product waste is environmentally hazardous but preventable. Therefore, it is important to understand tobacco disposal behaviors among those using tobacco products.

**Objectives:**

To explore self-reported disposal practices of cigarette butts and electronic nicotine products (ENP) components among adults (aged 18+).

**Methods:**

We used nationally representative cross-sectional data from Wave 6 (2021; n = 29,516) of the Population Assessment of Tobacco and Health (PATH) Study among adults who used cigarettes (manufactured and/or roll-your-own) and/or ENP every day, some days, or in the past 30 days.

**Results:**

In 2021, 89.7% (95% CI: 88.7, 90.6) of adults who smoked manufactured cigarettes usually disposed of cigarette butts in landfills (in an ash tray, in a cigarette disposal, or in the trash). Most adults who usually disposed of butts in landfills smoked daily (73.1%; 95% CI: 71.3, 74.9) and smoked an average of 14.6 cigarettes per day. Among those who used ENP, most adults usually disposed of the components in landfills (disposable devices: 83.1%; 95% CI: 80.5, 85.4; empty pods and cartridges: 85.4%; 95% CI: 82.4, 87.9; coils and atomizers: 71.2%; 95% CI: 68.1, 74.1; batteries: 48.5%; 95% CI: 45.2, 51.9; e-liquid containers: 70.9%; 95% CI: 67.1, 74.5; and leftover or unused e-liquid: 50.5%; 95% CI: 45.8, 55.2). Recycling as a usual practice was limited for people who used ENP- for disposable devices: 6.0% (95%CI: 4.6, 7.8); empty pods and cartridges: 5.0% (95% CI: 3.7, 6.8); coils and atomizers: 11.7% (95% CI: 9.4, 14.5); batteries: 23.7% (95% CI: 20.9, 26.8); and e-liquid containers: 18.0% (95% CI: 15.1, 21.3).

**Discussion:**

These findings demonstrate the large scope of tobacco product waste disposal in the United States and may inform efforts to address tobacco product waste management, such as environmental impact assessments and consumer education about proper disposal of tobacco products.

## Introduction

In 2019, globally, an estimated 1.14 billion people consumed 7.41 trillion cigarette-equivalents of tobacco [1]. Cigarette butts constitute the most commonly littered item, comprising 30–40% of all items picked up in annual international and United States (U.S.) beach cleanups since the 1980s [2–4]. Littering tobacco waste is rampant; people who smoke or use electronic nicotine products (ENP) generally have limited knowledge about the toxicity of tobacco waste or of the constituents of cigarette butts and ENP components [4–6]. The environmental impact of cigarettes can be divided into phases of tobacco growing and curing, manufacturing, use, and tobacco waste post consumption [7]. Tobacco growing involves use of chemicals including pesticides and fertilizers, which may affect drinking water sources and deplete soil nutrients [8]. In addition, tobacco manufacturing may produce solid waste, nicotine waste, and other toxic by-products such as ammonia [9,10], and nicotine and nicotine salts [9] as the most commonly-reported, while previously reported emissions of toluene, and methyl ethyl ketone are now rarely observed [7]. Toxic chemicals, including nicotine, polycyclic aromatic hydrocarbons (PAHs), and heavy metals, such as arsenic, cadmium, and lead may leach into the environment from littered cigarette butts, causing potential damage to ecosystems [11–13]. However, cigarettes are not listed as hazardous waste because the nicotine in them is not considered a commercial chemical product [14]. Additionally, cigarette butts are made of non-biodegradable plastic cellulose acetate fibers that can persist in the environment for more than 18 months and up to 30 years [13,15,16]. Cellulose acetate fibers are one of the most common plastic pollutants, and if not disposed of correctly, may continuously release microplastics into the environment contaminating aquatic environments and affecting wildlife [15,17]. Each cigarette butt contains more than 15,000 separate strands of plastic fibers [15,18] and smoked discarded butts release about 300,000 tons of potential microplastic fibers into the aquatic ecosystem [15,17,19]. This makes discarded cigarette butts an enduring environmental problem and the plastics and chemicals in them can bioaccumulate in aquatic organisms, potentially impacting the human food chain [20].

While there are many laboratory studies on the hazardous effects of cigarette butt waste [7,21–28], very little is known about the environmental hazards posed by improper disposal of ENP and their components. Some recent studies have attempted to identify the environmental impacts of ENPs [29–31]. Concerns arise regarding the disposal of ENP waste because of the high levels of copper, a potentially cytotoxic metal, as well as other metals, battery acids, nicotine, and other organic chemicals from ENP components [28,32,33]. The U.S. Environmental Protection Agency (EPA) has classified ENP waste as hazardous, and when collected in quantity, it must be disposed of appropriately [32,34]. As of February 2025, the U.S. EPA recommends not disposing ENPs in household trash or recycling but disposing at household hazardous waste collection sites [35]. However, additional research is needed to understand the potential public health challenge represented by ENP waste disposal, including understanding how people who use cigarettes and/or ENP are disposing of these products. Using the Population Assessment of Tobacco and Health (PATH) Study Wave 6 data (2021), the primary goal of this study is to explore disposal practices among people who use cigarettes and ENP in the U.S.

### Methods study design, setting, and participants

The PATH Study is an ongoing, nationally representative, longitudinal cohort study of adults and youth in the U.S. that collects information on tobacco use patterns and associated health behaviors. The PATH Study recruitment for the Wave 1 Cohort (2013) employed a stratified address-based, area-probability sampling design that oversampled adults who use tobacco, young adults (aged 18–24), and African American adults. For Wave 4, a probability replenishment sample was selected from the U.S. civilian non-institutionalized population (CNP) (data collected from December 1, 2016, through January 3, 2018), including persons who were not in the CNP at the time of Wave 1 (such as recent immigrants or those returning home from deployment). Members of the Wave 1 Cohort who remained in the CNP at the time of Wave 4 were combined with the Wave 4 replenishment sample to form the new Wave 4 Cohort. Details on interview procedures, questionnaires, sampling, weighting, response rates, and accessing the data are described in the *PATH Study Restricted Use Files User Guide* at https://doi.org/10.3886/Series606.

This study analyzed Wave 4 Cohort adult (aged ≥ 18) data from Wave 6 (2021; n = 29,516) of the PATH Study. Due to the ongoing COVID-19 pandemic, Wave 6 (data collected from youth and adults aged ≥ 14 from March 1, 2021, to November 30, 2021) was the first wave for which both telephone and in-person data collection methods were used. In-person collection was prioritized over telephone data collection, where permitted by each local jurisdiction’s public health COVID-19 guidelines and participant comfort. At Wave 6, the weighted response rate for adults for the Wave 4 Cohort, conditional upon Wave 4 participation, was 73.5%.

Full-sample and replicate weights were created to adjust for the complex sample design (e.g., oversampling) and nonresponse. Weighted estimates from Wave 6 represent the U.S. resident population (aged ≥ 14) at the time of Wave 6 who were part of the U.S. CNP at Wave 4. The replicate weights enable computation of associated measures of statistical precision. This analysis used the Wave 6 single-wave weights for Wave 4 Cohort to obtain statistically valid estimates from cross-sectional analyses. Further details regarding the PATH Study design and methods, as well as reliability and validity of responses are published elsewhere [25–27]. Missing data on sex, race and, ethnicity were imputed as described in the PATH Study RUF User Guide. Missing data on tobacco use disposal items was very low (<2%) and those missing on these items were not included in the analyses. The study was conducted by Westat and approved by the Westat Institutional Review Board (IRB). All adults aged ≥ 18 provided written (in-person) or verbal (over the telephone) informed consent that was approved by the IRB. Appendix A presents the Preferred Reporting Items for Complex Sample Survey Analysis (PRICSSA) for the current study.

### Measures

*Tobacco product use:* The PATH Study questionnaire asks about the use of cigarettes, ENP, and other tobacco products. All participants who report ever using tobacco products are asked about their current product use (every day, some days, or past 30-day use). For cigarettes, people who currently smoke are asked if they use manufactured, roll-your-own (RYO), or both cigarette types. At Wave 6, ENP were described in the PATH Study questionnaire as “electronic nicotine products such as e-cigarettes, pod devices, vape pens, tank systems, mods, e-cigars, e-pipes, e-hookahs, and hookah pens” that “are battery-powered and produce vapor instead of smoke. They typically use a nicotine liquid called ‘e-liquid,’ although the amount of nicotine can vary, and some may not contain any nicotine at all. Some common brands include JUUL, Vuse, Blu, NJOY, eGo, Suorin, Bo, Smok, Phix, and Puff Bar, but there are many others.” ENP device types were categorized as follows in the PATH Study questionnaire: (1) disposable devices, (2) devices that use replaceable pods or cartridges, (3) devices with a tank that is refilled with liquids, (4) mod systems, or (5) something else. Use of other tobacco products was defined as current (every day, some days, or past 30-days) use of traditional cigars, cigarillos, filtered cigars, pipe tobacco, hookah, smokeless tobacco, or snus. Dual use was defined as current use of cigarettes and ENP, irrespective of the use of other tobacco products. Current cigarette or ENP use was defined as daily or non-daily use based on current use (every day, some days, or past 30-day) and the number of days used in the past 30 days. For daily use, cigarettes per day were defined as self-reported cigarettes smoked per day. For non-daily use, cigarettes per day was the average number of cigarettes smoked per day, using the number of cigarettes usually smoked per day on days smoked in the past 30 days and the number of days smoked in the past 30 days. For example, a person who smoked non-daily on 12 of the past 30 days and smoked 10 cigarettes per day on those 12 days, smoked an average of four cigarettes per day in the past 30 days (12 days smoked X 10 cigarettes per day on days smoked = 120 cigarettes/30 days = 4 cigarettes per day in the past 30 days).

#### Disposal practices.

Cigarettes: Adults who reported current use of manufactured cigarettes were asked about their disposal practices of cigarette butts with mutually exclusive and write-in options. Adults who currently used manufactured and RYO cigarettes were then asked what they usually do with a cigarette pack, carton, or RYO pouch when it is empty, and they were given response options and write-in options if they chose a practice that was not listed in the response options. All write-in options were independently reviewed by an analyst and were either categorized into existing categories or assigned new categories to capture all responses. A second analyst reviewed the recodes independently and any disagreements between the two analysts were resolved by a third analyst after discussions until a consensus was reached. Recodes of write-in responses are shown in S1 Table. Details of questions asked, response options, and final recodes are explained in Appendix B. Final response options were recoded into and hereafter referred to as (1) Landfill (e.g., “in an ash tray or cigarette disposal,” “in the trash”), (2) Litter/Sewer (e.g., “on the ground”, “out of a car window”, and “throw in sink or toilet”), (3) Container (e.g., “can”, “bottle”), or (4) Other (e.g., “fire pit”) based on the frequency distribution of the response options. The “Other” category served as a catch-all for responses that did not fit in the other categories and ambiguous responses such as “anywhere” were included in the “Other” category as a conservative approach. Those who chose “don’t know” were included in the “Other” category. Those who selected “refused” or responded that they were not using cigarettes were set to missing for analyses.

ENP: Adults currently using ENP were asked several questions on product and component disposal based on the device types they used. Six separate questions were asked on disposal practices of disposable ENP, prefilled pods or cartridges (among those who use prefilled pods or cartridges, refillable, or a mod system device), atomizers after they no longer work (among those who use refillable devices or a mod system devices), bottle or container of e-liquid or leftover or unused e-liquid (among those who use refillable devices) with mutually exclusive and write-in options if they chose a practice not listed in the response options. Like cigarettes, all write-in options were closely reviewed by analysts and were either categorized into existing categories or assigned new categories to capture all responses (see S2–S7 Tables). Final response options included (1) Landfill (e.g., “throw it away in the trash”), (2) Litter/Sewer (e.g., “throw it on the ground”), (3) Recycle/Return/Reuse (e.g., “return it to store/vape shop”), (4) Have not gotten rid of one, and (5) Other (e.g., “give it back to someone else”). Those who chose “don’t know” were included in the “Other” category. Those who selected the “refused” option or responded that they were not using ENPs were set to missing for analyses (see Appendix B for details).

Other characteristics: Sociodemographic variables included age (18 –24 ; 25 –39 ; 40 –54 ; ≥  55), sex (male; female), race/ethnicity (non-Hispanic White; non-Hispanic Black; non-Hispanic other; Hispanic), education (less than high school; General Education Development (GED); high school graduate; some college; college or more), and annual family income (<$25,000; $25,000-$74,999; ≥ $75,000; not reported).

#### Statistical analyses.

All analyses were conducted using SAS Survey Procedures, V.9.4 (SAS Institute). Variances were estimated using the balanced repeated replication method [36] with Fay’s adjustment set to 0.3 to increase estimate stability [37]. Analyses were run on the Wave 6 Restricted Use Files (https://doi.org/10.3886/ICPSR36498.v8). Estimates with low precision (those based on fewer than 50 observations in the denominator or the coefficient of variation of the estimate or its complement is larger than 30% with a relative standard error greater than 0.30) were flagged and are not discussed in the results. To explore the potential impacts of protocol changes at Wave 6, sensitivity analyses were conducted exploring differences in responses among adults who were interviewed in-person and by telephone.

## Results

In Wave 6, 15.7% (n = 6,333), 1.4% (n = 559), and 7.5% (n = 3,913) of adults currently used manufactured cigarettes, RYO cigarettes, and ENP, respectively. Demographic characteristics of adults who currently use cigarettes and/or ENP are presented in Table 1.

**Table 1:** Demographic and Tobacco Use Patterns Among Adults Who Currently Use Cigarettes and ENP, Wave 6 (2021) of the PATH Study.

	Current Manufactured Cigarette^a^ n = 6,333; 15.7% (95% CI: 15.2–16.2)		Current Roll-Your-Own Cigarette^b^ n = 559; 1.4% (95% CI: 1.2–1.6)		Current ENP n = 3,913; 7.5% (95% CI: 7.2–7.8)	
**Age Groups**	**Unweighted n/mean (SE)**	**Weighted %** **(95% CI)**	**Unweighted n/mean (SE)**	**Weighted %** **(95% CI)**	**Unweighted n/mean (SE)**	**Weighted %** **(95% CI)**
18-24	1,018	8.3 (7.6-9.0)	83	8.0 (5.9-10.7)	1,989	32.5 (30.9-34.2)
25-39	2,123	32.7 (31.4-34.0)	157	27.9 (24.1-32.0)	1,263	40.6 (38.7-42.5)
40-54	1,480	27.3 (25.6-29.1)	141	28.5 (24.1-33.4)	412	16.0 (14.5-17.6)
55+	1,712	31.7 (30.1-33.4)	178	35.6 (30.3-41.2)	249	10.9 (9.2-12.9)
**Sex**						
Male	3,048	54.0 (52.5-55.5)	306	62.0 (57.9-66.0)	1,928	52.8 (51.1-54.6)
Female	3,281	46.0 (44.5-47.5)	252	38.0 (34.0-42.1)	1,982	47.2 (45.4-48.9)
**Race/ethnicity**						
Non-Hispanic White	3,702	65.2 (63.7-66.6)	394	78.7 (74.3-82.5)	2,302	66.8 (64.7-68.9)
Non-Hispanic Black	1,031	14.4 (13.5-15.4)	61	9.4 (7.0-12.4)	399	9.9 (8.7-11.2)
Non-Hispanic Other/Multiple	444	5.6 (4.9-6.4)	40	4.8 (3.1-7.4)	372	7.6 (6.7-8.6)
Hispanic	1,022	14.8 (13.8-15.9)	51	7.1 (4.8-10.5)	786	15.6 (14.3-17.1)
**Education**						
Less than high school/some high school/no diploma	997	15.0 (13.9-16.2)	128	21.7 (17.9-25.9)	414	10.3 (8.9-11.8)
GED	614	9.9 (9.1-10.9)	74	12.6 (9.7-16.3)	225	6.6 (5.4-8.1)
High school graduate	1,702	29.6 (28.1-31.2)	149	33.6 (28.5-39.1)	1,117	27.9 (25.8-30.2)
Some college/2-year degree	2,229	32.9 (31.4-34.4)	158	25.0 (21.3-29.2)	1,606	39.9 (37.8-42.0)
College or more	764	12.5 (11.4-13.8)	46	7.1 (5.2-9.5)	537	15.2 (13.8-16.8)
**Income**						
< 25,000	2,461	36.5 (34.9-38.1)	304	54.1 (48.9-59.2)	1,188	30.5 (28.6-32.5)
25,000-74,999	2,373	38.8 (37.4-40.2)	168	30.7 (26.1-35.7)	1,446	37.6 (35.7-39.6)
75,000+	1,180	20.2 (18.6-22.0)	51	9.2 (6.7-12.5)	1,013	26.5 (24.3-28.7)
Not reported	319	4.5 (3.9-5.2)	36	6.0 (3.9-9.0)	266	5.4 (4.6-6.2)
**Use of other tobacco products** ^**c**^	1,463	21.9 (20.5-23.3)	184	32.8 (27.9-38.1)	1,099	29.1 (27.3-30.9)
**Dual use of cigarettes and ENP**	1,581	21.0 (19.8-22.2)	145	23.3 (19.2-28.0)	1,641	45.9 (43.8-48.0)
**Frequency of past 30-day or current use**						
Daily	4,316	71.2 (69.6-72.8)	432	81.4 (77.9-84.5)	1,640	43.5 (41.3-45.6)
Non-daily	2,037	28.8 (27.2-30.4)	126	18.6 (15.5-22.1)	2,267	56.5 (54.4-58.7)
**ENP Device types**						
Disposable	668	41.7 (38.2-45.3)	55	35.7 (24.7-48.5)	1,690	40.7 (38.3-43.2)
Replaceable pods or cartridges	331	21.0 (18.7-23.4)	30	18.6 (12.5-26.6)	798	20.9 (19.2-22.7)
Refillable tank	448	31.3 (28.1-34.6)	43	36.9 (27.1-48.0)	1,109	31.8 (29.5-34.3)
Mod system	60	3.6 (2.6-5.0)	12	7.6† (3.5-16.0)	168	4.6 (3.7-5.6)
Something else	30	2.4 (1.4-4.0)	3	1.2† (0.3-4.6)	63	2.0 (1.4-2.8)
**Types of cigarettes smoked**						
Manufactured	5,885	92.9 (92.0-93.7)	NA	NA	1,422	86.9 (84.4-89.1)
Roll-your-own (RYO)	NA	NA	282	52.4 (47.2-57.5)	53	3.6 (2.7-4.9)
Both	277	4.3 (3.7-4.9)	277	47.6 (42.5-52.8)	92	5.9 (4.4-8.0)
I don’t know	171	2.9 (2.4-3.5)	NA	NA	67	3.6 (2.6-4.8)
**Cigarettes per day (mean, ± SE)**	10.7 ± 0.2	(10.3-11)	14.9 ± 0.6	(13.6-16.1)	7.8 ± 0.3^**d**^	(7.2-8.4)
Daily	14.5 ± 0.2	(14.1-14.8)	17.9 ± 0.6	(16.6-19.2)	13.7 ± 0.4	(13.0-14.5)
Non-daily	1.2 ± 0.1	(1.1-1.4)	1.6 ± 0.4	(0.9-2.3)	1.1 ± 0.2	(0.8-1.4)

Percentages are weighted using Wave 6 single-wave weights. Ns are unweighted. The total PATH Study Wave 6 sample includes 29,516 participants aged ≥18 years. Ns in this table may add up to less than the totals due to missing data. CI, confidence interval; ENP, electronic nicotine products (commonly: e-cigarettes); PATH, Population Assessment of Tobacco and Health; NA, not applicable. †Estimate should be interpreted with caution because there is low statistical precision. Estimate is based on a denominator sample size of less than 50, or the coefficient of variation of the estimate or its complement is larger than 30%.

^a^Current manufactured cigarette use excludes exclusive use of roll-your-own tobacco. It is not mutually exclusive with roll-your-own cigarette use.

^b^Current Roll-Your-Own cigarette use includes use of roll-your-own tobacco exclusively and both roll-your-own tobacco and manufactured cigarettes.

^c^Other tobacco definition includes traditional cigars, cigarillos, filtered cigars, pipe tobacco, hookah, smokeless tobacco, or snus.

^d^Among respondents who currently used cigarettes and ENP or have used in the past 30 days.

### Usual disposal practices of cigarette butts and RYO tobacco pouches

**Fig 1:**
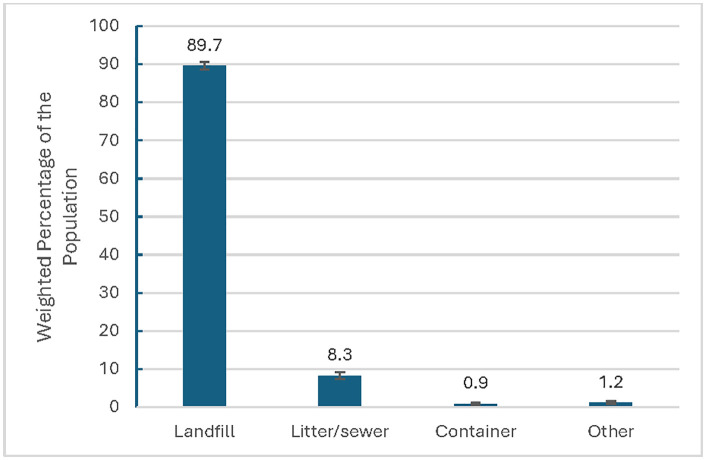
Cigarette Butt Disposal Practices Among Adults Who Currently Use Cigarettes, Wave 6 (2021) of the PATH Study. Landfill (n = 5,646) includes disposal in an ashtray, cigarette disposal or trash; litter/sewer (n = 565) includes disposal on the ground, in the sink or toilet, or out of a car window; container (n = 55) includes disposal in cans, bottles, etc; other (n = 65) includes disposal in fire pits, somewhere else, etc.

About 90% (89.7%; 95% CI: 88.7, 90.6) of adults who currently smoked cigarettes usually disposed of their cigarette butts in landfills (i.e., ash tray, cigarette disposal container, or in the trash), followed by 8.3% (95% CI: 7.4, 9.2) who usually disposed of butts as litter or in the sewer and few (0.9%; 95% CI: 0.6, 1.2) usually discarded their butts into containers (e.g., cans, bottles) (Fig 1). Among those who currently smoke cigarettes and usually littered (weighted % = 8.3; weighted sample size N = 3,216,993 and whose weighted average cigarettes smoked per day among those who littered was 7.8; Table 2), an estimated 9.2 billion cigarette butts were littered in 2021 (3,216,993 (weighted sample size) X 7.8 cigarettes per day X 365 days = 9.2 billion). Adults who usually disposed of their cigarette butts in landfills were predominantly (32.9%) aged ≥ 55 (95% CI: 31.2, 34.6) or aged 25–39 (32.0%; 95% CI: 30.6, 33.5; Table 2). Of the adults who usually littered their cigarette butts, 43.1% were adults aged 25–39 (95% CI: 37.9, 48.6). For both methods of disposal, more than half were male (landfill: 52.9%; 95% CI: 51.3, 54.5; litter: 64.9%; 95% CI: 59.3, 70.1), non-Hispanic White (landfill: 67.1%; 95% CI: 65.5, 68.7; litter: 44%; 95% CI: 38.7, 49.5), and had some college education (landfill: 32.7%; 95% CI: 31.1, 34.3; litter: 34.8%; 95% CI: 30.3, 39.6). Those who usually disposed of butts in landfills predominately smoked daily (73.1%; 95% CI: 71.3, 74.9) with an average of 14.6 cigarettes per day (95% CI: 14.2, 14.9; see Table 2).

**Table 2:** Cigarette Butt Disposal Practices by Sociodemographic and Tobacco Use Characteristics Among Adults Who Currently Use Cigarettes, Wave 6 (2021) of the PATH Study.

	Current cigarette use (n = 6331)							
	Landfill n = 5,646; 89.7% (95% CI: 88.7–90.6)		Litter/Sewer n = 565; 8.3% (95% CI: 7.4–9.2)		Container n = 55; 0.9% (95% CI: 0.6–1.2)		Other n = 65; 1.2% (95% CI: 0.9–1.6)	
**Age Groups**	**Unweighted n/mean (SE)**	**Weighted %** **(95% CI)**	**Unweighted n/mean (SE)**	**Weighted %** **(95% CI)**	**Unweighted n/mean (SE)**	**Weighted %** **(95% CI)**	**Unweighted n/mean (SE)**	**Weighted %** **(95% CI)**
18-24	825	7.5 (6.9-8.2)	166	15.9 (13.1-19.1)	5	4.1† (1.2-13.0)	17	11.4† (5.7-21.3)
25-39	1,862	32.0 (30.6-33.5)	231	43.1 (37.9-48.4)	16	22.9 (12.8-37.5)	16	20.3 (11.3-33.8)
40-54	1,363	27.6 (25.8-29.5)	90	24.3 (19.2-30.2)	14	28.9 (16.9-44.7)	15	24.9† (12.4-43.7)
55+	1,596	32.9 (31.2-34.6)	78	16.7 (13.1-21.1)	20	44.2 (27.9-61.8)	17	43.4 (24.5-64.5)
**Sex**								
Male	2,650	52.9 (51.3-54.5)	338	64.9 (59.3-70.1)	26	45.1 (31.2-59.9)	35	64.8 (48.1-78.5)
Female	2,993	47.1 (45.5-48.7)	226	35.1 (29.9-40.7)	29	54.9 (40.1-68.8)	30	35.2 (21.5-51.9)
**Race/ethnicity**								
Non-Hispanic White	3,393	67.1 (65.5-68.7)	229	44.0 (38.7-49.5)	38	65.0 (44.4-81.1)	37	61.4 (41.8-77.8)
Non-Hispanic Black	911	14.1 (13.1-15.3)	111	19.9 (16.2-24.2)	3	2.6† (0.6-10.9)	10	11.2† (5.6-21.2)
Non-Hispanic Other/Multiple	391	5.3 (4.6-6.0)	44	8.5 (5.7-12.5)	4	16.4† (5.6-39.2)	5	4.5† (1.3-14.8)
Hispanic	830	13.5 (12.3-14.7)	170	27.5 (22.9-32.7)	9	16.1† (8.6-28.1)	12	22.9† (9.6-45.3)
**Education**								
Less than high school/some high school/no diploma/GED	1,439	24.9 (23.4-26.4)	143	25.1 (20.5-30.2)	10	18.4 (10.3-30.6)	15	23.5†(12.3-40.2)
High school graduate	1,514	29.8 (28.2-31.5)	161	29.1 (24.3-34.3)	14	28.6 (16.4-45.0)	12	24.0† (11.7-43.0)
Some college/2-year degree	1,986	32.7 (31.1-34.3)	198	34.8 (30.3-39.6)	24	37.9 (25.3-52.2)	25	34.8 (20.1-53.0)
College or more	687	12.6 (11.3-14.0)	59	11.1 (8.0-15.1)	7	15.2† (6.1-33.0)	10	17.7† (6.8-39.0)
**Income**								
< 25,000	2,201	36.6 (35.0-38.3)	217	34.9 (30.1-40.0)	17	35.9 (23.4-50.7)	22	32.7 (18.1-51.8)
25,000-74,999	2,130	39.0 (37.5-40.5)	205	38.5 (33.7-43.4)	21	35.5 (22.8-50.8)	19	35.9 (19.1-57.1)
75,000+	1,041	20.0 (18.3-21.7)	111	21.5 (16.6-27.5)	12	22.5† (11.7-39.1)	17	25.5 (14.1-41.7)
Not reported	274	4.4 (3.8-5.1)	32	5.1 (3.3-8.0)	5	6.0† (2.1-16.2)	7	5.9† (2.0-15.7)
**Use of other tobacco products** ^a^	1,241	20.7 (19.3-22.1)	193	34.0 (28.3-40.1)	10	16.4† (8.5-29.3)	23	38.2 (22.0-57.6)
**Dual cigarette and ENP use**	1,342	20.1 (19.0-21.3)	203	29.3 (24.9-34.2)	10	13.4† (6.1-26.8)	22	23.6† (11.9-41.2)
**Frequency of past 30-day or current use**								
Daily	3,981	73.1 (71.3-74.9)	274	55.5 (50.5-60.4)	36	69.9 (52.9-82.8)	19	29.2 (16.2-46.7)
Non-daily	1,660	26.9 (25.1-28.7)	291	44.5 (39.6-49.5)	19	30.1 (17.2-47.1)	44	70.8 (53.3-83.8)
**Cigarettes per day (mean, ± SE)**	11 ± 0.2	(10.6-11.4)	7.8 ± 0.5	(6.8-8.8)	9.8 ± 1.1	(7.7-12)	3.4 ± 0.9	(1.6-5.2)
Daily	14.6 ± 0.2	(14.2-14.9)	13.3 ± 0.6	(12-14.5)	13.3 ± 1.1	(11.1-15.6)	11.3 ± 2	(7.4-15.3)
Non-daily	1.3 ± 0.1	(1.1-1.5)	0.9 ± 0.1	(0.6-1.2)	1.7 ± 0.6	(0.4-3)	0.2 ± 0.2	(−0.1-0.5)

Percentages are weighted using Wave 6 single-wave weights. Ns are unweighted. The total PATH Study Wave 6 sample includes 29,516 participants aged ≥18 years. Ns in this table may add up to less than the totals due to missing data. CI, confidence interval; ENP, electronic nicotine products (commonly: e-cigarettes); PATH, Population Assessment of Tobacco and Health; landfill includes disposal in an ashtray, cigarette disposal or trash; litter includes disposal on the ground, in the sink or toilet, or out of a car window; container includes disposal in cans, bottles, etc; other includes disposal in fire pits, somewhere else, etc. Estimates are collapsed due to small sample size (n = 1 or n = 2); for education, less than high school/some high school/no diploma was combined with GED.

^†^Estimate should be interpreted with caution because it has low statistical precision. The estimate is based on a denominator sample size of less than 50, or the coefficient of variation of the estimate or its complement is larger than 30%.

^a^Other tobacco definition includes traditional cigars, cigarillos, filtered cigars, pipe tobacco, hookah, smokeless tobacco, or snus.

**Fig 2:**
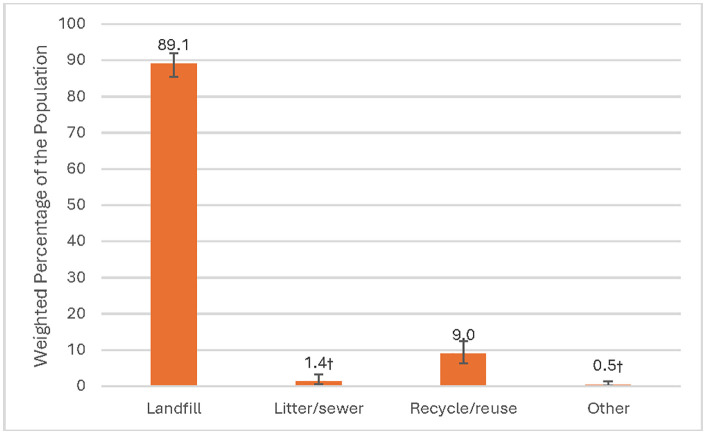
Roll-Your-Own Tobacco Pouch Disposal Practices Among Adults Who Currently Smoke Roll-Your-Own Cigarettes, Wave 6 (2021) of the PATH Study. † Estimate should be interpreted with caution because it has low statistical precision. The estimate is based on a denominator sample size of less than 50, or the coefficient of variation of the estimate or its complement is larger than 30%. Landfill (n = 485) includes disposal in an ashtray, cigarette disposal or trash; litter/sewer (n = 9) includes disposal on the ground, in the sink or toilet, or out of a car window; other (n = 5) includes burning; recycle/reuse (n = 50).

Among adults who smoked RYO cigarettes, 89.1% (95% CI: 85.6, 91.9) disposed of their tobacco pouches in landfills, followed by 9% (95% CI: 6.1, 12.4) who recycled or reused (Fig 2). Of the adults that disposed of their tobacco pouches in landfills, the majority (37.6%) were aged ≥ 55 (95% CI: 32.0, 43.6), male (62.3%, 95% CI: 57.7, 66.7), non-Hispanic White (79.8%, 95% CI: 74.6, 84.2), and smoked daily (82.0%, 95% CI: 77.8, 85.5) with an average of 18 cigarettes per day (95% CI: 16.7, 19.4; see Table 3).

**Table 3:** Roll-Your-Own Tobacco Pouch Disposal Practices by Sociodemographic and Tobacco Use Characteristics Among Adults Who Currently Smoke Roll-Your-Own Cigarettes, Wave 6 (2021) of the PATH Study.

	Current roll-your-own cigarette use (n = 549)			
	Landfill n = 485; 89.1% (95% CI: 85.6–91.9)		Recycle/Reuse n = 50; 9% (95% CI: 6.4–12.4)	
**Age Groups**	**Unweighted n/mean (SE)**	**Weighted %** **(95% CI)**	**Unweighted n/mean (SE)**	**Weighted %** **(95% CI)**
18-24	69	7.8 (5.6-10.9)	7	5.9† (2.0-16.0)
25-39	129	26.5 (22.6-30.9)	19	35.1 (21.3-51.9)
40-54	123	28.1 (23.4-33.2)	13	34.2 (19.8-52.3)
55+	164	37.6 (32.0-43.6)	11	24.8 (13.8-40.5)
**Sex**				
Male	266	62.3 (57.7-66.7)	29	61.0 (46.1-74.1)
Female	218	37.7 (33.3-42.3)	21	39.0 (25.9-53.9)
**Race/ethnicity**				
Non-Hispanic White	352	79.8 (74.6-84.2)	31	73.8 (60.2-83.9)
Non-Hispanic Black	52	9.3 (6.7-12.9)	4	6.0† (2.1-16.3)
Non-Hispanic Other/Multiple	32	4.5 (2.7-7.5)	7	7.9† (3.1-18.7)
Hispanic	38	6.3 (3.9-10.1)	8	12.3† (5.0-27.1)
**Education**				
Less than high school/some high school/no diploma	117	21.8 (18.0-26.1)	7	22.0† (10.5-40.3)
GED	62	12.1 (9.0-16.0)	7	13.8† (6.4-27.1)
High school graduate	136	35.5 (30.1-41.3)	9	18.6† (8.6-35.7)
Some college/2-year degree	132	24.1 (20.0-28.8)	16	31.9 (18.7-48.8)
College or more	36	6.5 (4.6-9.1)	9	13.6† (6.5-26.3)
**Income**				
< 25,000	264	54.4 (49.1-59.6)	26	48.2 (32.8-63.9)
25,000-74,999	149	30.9 (26.0-36.4)	14	31.1 (19.4-45.9)
75,000 + /Not reported	72	14.7 (11.4-18.7)	10	20.7† (10.7-36.3)
**Use of other tobacco products** ^**a**^	149	31.1 (26.0-36.7)	22	42.8 (28.2-58.8)
**Dual cigarette and ENP use**	121	21.8 (17.6-26.7)	14	30.5 (17.2-48.3)
**Frequency of past 30-day or current use**				
Daily	381	82.0 (77.8-85.5)	35	77.4 (63.7-87.0)
Non-daily	104	18.0 (14.5-22.2)	15	22.6 (13.0-36.3)
**Cigarettes per day (mean, ± SE)**				
Daily	18 ± 0.7	(16.7-19.4)	15.7 ± 1.3	(13.1-18.4)
Non-daily	1.7 ± 0.4	(0.9-2.5)	1 ± 0.4	(0.2-1.9)

Percentages are weighted using Wave 6 single-wave weights. Ns are unweighted. The total PATH Study Wave 6 sample includes 29,516 participants aged ≥18 years. Ns in this table may add up to less than the totals due to missing data. CI, confidence interval; ENP, electronic nicotine products (commonly: e-cigarettes); PATH, Population Assessment of Tobacco and Health; landfill includes disposal in an ashtray, cigarette disposal or trash; litter includes disposal on the ground, in the sink or toilet, or out of a car window; other includes burning. Litter disposal method n = 9; data are not presented in the table due to small n. Other disposal method n = 5, data are not presented in the table due to small n. Estimates are collapsed due to small sample size (n = 1 or n = 2); for income, “not reported” was combined with $75,000 and up.

^†^Estimate should be interpreted with caution because it has low statistical precision. The estimate is based on a denominator sample size of less than 50, or the coefficient of variation of the estimate or its complement is larger than 30%.

^a^Other tobacco definition includes traditional cigars, cigarillos, filtered cigars, pipe tobacco, hookah, smokeless tobacco, or snus.

### Usual disposal practices of ENP components

#### Disposable devices.

Of adults using disposable ENP; 83.1% (95% CI: 80.5, 85.4) usually discarded their devices in landfills, and only 6.0% (95% CI: 4.6, 7.8) usually recycled, returned, or reused their disposable ENP (Table 4). Those who usually discarded their disposable ENP in landfills were most commonly young adults aged 18–24 (39.9%; 95% CI: 36.5, 43.4), were females (50.2%; 95% CI: 46.2, 54.2), and had some college education (39.9%; 95% CI: 36.1, 43.7). Those who usually recycled their disposable ENP were most commonly adults aged 25–39 (45.1%; 95% CI: 30.5, 60.7), and were males (61.3%; 95% CI: 46.8, 73.9). Adults in both groups had similar tobacco product use characteristics. More than 40% had dual use of ENP and cigarettes (landfill: 48.0%; 95% CI: 44.6, 51.5; recycle: 46.7%; 95% CI: 33.7, 60.1), and the majority used ENP non-daily (landfill: 63.0%; 95% CI: 59.6, 66.2; recycle: 71.3%; 95% CI: 59.0, 81.1).

**Table 4:** Disposal Practices of Disposable ENP Device and Empty Pod or Cartridge Among Adults Who Currently Use ENP, Wave 6 (2021) of the PATH Study.

	Disposable device (n = 1,685)			Empty pod or cartridge (among users of devices with replaceable pods or cartridges; n = 791)		
	Landfill n = 1,394; 83.1% (95% CI: 80.5–85.4)	Recycle/return/reuse n = 94; 6% (95% CI: 4.6–7.8)	Have not gotten rid of an empty one^a^ n = 177; 9.5% (95% CI: 7.8–11.5)	Landfill n = 659; 85.4% (95% CI: 82.4–87.9)	Recycle/return/reuse n = 36; 5% (95% CI: 3.7–6.8)	Have not gotten rid of an empty one n = 93; 9.4% (95% CI: 7.4–11.8)^a^
**Age Groups**	**Weighted %** **(95% CI)**	**Weighted %** **(95% CI)**	**Weighted %** **(95% CI)**	**Weighted %** **(95% CI)**	**Weighted %** **(95% CI)**	**Weighted %** **(95% CI)**
18-24	39.9 (36.5-43.4)	41.6 (29.3-55.0)	51.9 (42.5-61.2)	33.1 (29.2-37.3)	19.2† (9.6-34.8)	54.7 (43.2-65.8)
25-39	37.9 (34.4-41.5)	45.1 (30.5-60.7)	38.9 (29.6-48.9)	36.4 (32.1-41.0)	49.5† (31.7-67.4)	38.1 (26.2-51.5)
40+	22.2 (18.8-26.1)	13.3† (6.6-25.0)	9.2 (5.3-15.7)	30.5 (26.0-35.3)	31.3† (18.5-47.8)	7.2† (2.8-17.6)
**Sex**						
Male	49.8 (45.8-53.8)	61.3 (46.8-73.9)	39.0 (30.1-48.7)	55.9 (51.1-60.6)	63.0† (43.4-79.1)	37.1 (28.0-47.2)
Female	50.2 (46.2-54.2)	38.7 (26.1-53.2)	61.0 (51.3-69.9)	44.1 (39.4-48.9)	37.0† (20.9-56.6)	62.9 (52.8-72.0)
**Race/ethnicity**						
Non-Hispanic White	61.4 (57.5-65.2)	50.1 (37.6-62.5)	61.5 (51.4-70.8)	73.7 (68.9-78.1)	61.2† (41.4-77.9)	62.8 (49.7-74.3)
Non-Hispanic Black	12.2 (10.2-14.5)	6.9† (3.3-13.9)	12.4† (6.8-21.7)	6.4 (4.4-9.2)	14.9† (4.6-39.0)	13.7† (7.2-24.4)
Non-Hispanic Other/Multiple	8.3 (6.9-9.9)	9.7† (4.0-21.7)	9.1 (5.2-15.5)	7.3 (5.1-10.4)	11.4† (4.6-25.7)	8.5† (4.3-16.0)
Hispanic	18.1 (15.9-20.6)	33.4 (20.6-49.2)	16.9 (11.6-24.1)	12.6 (9.8-16.1)	12.4† (5.2-26.9)	15.0 (8.7-24.7)
**Education**						
Less than high school/some high school/no diploma/GED	18.0 (15.3-21.0)	30.6 (16.8-49.1)	8.7 (5.2-14.2)	13.1 (10.0-16.9)	15.4† (4.3-42.1)	8.5† (3.1-21.6)
High school graduate	30.3 (26.5-34.3)	29.0 (18.8-41.9)	22.0 (16.4-28.8)	24.3 (20.1-29.1)	18.9† (7.1-41.4)	16.6 (10.2-25.9)
Some college/2-year degree	39.9 (36.1-43.7)	23.5 (15.8-33.5)	39.6 (30.5-49.5)	42.4 (38.1-46.8)	43.7† (25.3-64.1)	29.5 (20.9-39.9)
College or more	11.9 (10.2-13.8)	16.9† (8.8-29.9)	29.8 (21.1-40.1)	20.2 (16.6-24.3)	22.0† (9.7-42.5)	45.3 (34.3-56.9)
**Income**						
< 25,000	34.4 (31.0-38.0)	52.4 (39.7-64.8)	28.8 (21.2-37.8)	20.5 (17.0-24.5)	26.5† (14.2-43.9)	14.7 (8.2-24.8)
25,000-74,999	36.8 (32.7-41.0)	30.5 (21.8-40.8)	33.1 (25.1-42.2)	38.9 (34.1-43.9)	41.4† (23.8-61.4)	39.2 (28.1-51.5)
75,000 + /Not reported	28.8 (25.5-32.4)	17.1 (10.4-26.8)	38.1 (28.7-48.4)	40.6 (35.3-46.2)	32.2 (18.8-49.3)	46.2 (34.4-58.4)
**Dual cigarette and ENP use**	48.0 (44.6-51.5)	46.7 (33.7-60.1)	30.2 (22.4-39.3)	46.6 (41.9-51.4)	40.2† (22.7-60.6)	37.5 (25.6-51.1)
**Frequency of use**						
Daily	37.0 (33.8-40.4)	28.7 (18.9-41.0)	16.8 (11.3-24.2)	50.5 (45.5-55.6)	28.0† (14.6-47.1)	13.1 (7.5-21.9)
Non-daily	63.0 (59.6-66.2)	71.3 (59.0-81.1)	83.2 (75.8-88.7)	49.5 (44.4-54.5)	72.0† (52.9-85.4)	86.9 (78.1-92.5)

Percentages are weighted using Wave 6 single-wave weights. Ns are unweighted. The total PATH Study Wave 6 sample includes 29,516 participants aged ≥18 years. Ns in this table may add to less than the totals due to missing data. CI, confidence interval; ENP, electronic nicotine products (commonly: e-cigarettes); PATH, Population Assessment of Tobacco and Health; landfill includes disposal in the trash; litter includes disposal on the ground; recycle/return/reuse includes returning it to a store, etc; other includes giving them to someone else-. For disposal devices, litter disposal method N = 11 and other disposal method N = 9; data are not presented in the table due to small n. For empty pods or cartridges, litter disposal method N = 11 and other disposal method N = 9; data are not presented in the table due to small n. Estimates are collapsed due to small sample size (n = 1 or n = 2); ages 40 and up were collapsed into one category; for education, less than high school/some high school/no diploma was combined with GED; for income, “not reported” was combined with $75,000 and up.

†Estimate should be interpreted with caution because it has low statistical precision. The estimate is based on a denominator sample size of less than 50, or the coefficient of variation of the estimate or its complement is larger than 30%.

^a^Adults that indicated that they used someone else’s product were combined with those who had not gotten rid of an empty ENP device/pod or cartridge.

Columns on “littering” and “other” for empty pod or cartridge are not presented due to small sample sizes (n < 3).

#### Empty pods and cartridges.

Of those using pods or cartridge devices, 85.4% (95% CI: 82.4, 87.9) usually disposed of them in landfills, and only 5.0% (95% CI: 3.7, 6.8) usually recycled, returned, or reused their empty pods or cartridges. Adults in both groups had similar demographic characteristics (see Table 4). About half of those who usually disposed of pods and cartridges in landfills used ENP daily (50.5%; 95% CI: 45.5, 55.6).

#### Batteries.

Of those who used products requiring batteries, 48.5% (95% CI: 45.2, 51.9) usually disposed of their unneeded or nonfunctional batteries in landfills, and 23.7% (95% CI: 20.9, 26.8) usually recycled, returned, or reused their batteries (Table 5). Demographic characteristics were similar for those who usually disposed of batteries in landfills and those who recycled them. The largest share were adults aged 25–39 (landfill: 40.4%; 95% CI: 36.9, 44.1; recycle: 50.7%; 95% CI: 44.3, 57.2), male (landfill: 56.0%; 95% CI: 51.9, 60.1; recycle: 67.1%; 95% CI: 60.4, 73.2), and had some college education (landfill: 40.7%; 95% CI: 36.7, 44.9; recycle: 40.6%; 95% CI: 34.0, 47.5). Likewise, tobacco product use was similar among those who usually disposed of batteries in landfills or recycled them. Among those who used both cigarettes and ENP, slightly more discarded batteries in landfills (landfills: 47.0%; 95% CI: 42.4, 51.6) than recycled them (40.9%; 95% CI: 34.6, 47.5). About half of adults in each group used ENP daily (landfill: 55.5%; 95% CI: 50.9, 60.1; recycle: 57.5%; 95% CI: 50.7, 64.1).

**Table 5:** Disposal of Battery and Coil or Atomizer Among Adult Who Currently Use ENP, Wave 6 (2021) of the PATH Study.

	Battery (among users of devices with replaceable pods or cartridges, refillable tanks, mod systems, or something else; n = 1,542)				Coils or Atomizers (among users of devices with refillable tanks or mod systems; n = 1,269)		
	Landfill n = 768; 48.5% (95% CI: 45.2–51.9)	Recycle/Return/Reuse n = 343; 23.7% (95% CI: 20.9–26.8)	Have not gotten rid of an empty one^a^ n = 404; 26% (95% CI: 23–29.1)	Other n = 24; 1.6%† (95% CI: 0.8–3.1)	Landfill n = 903; 71.2% (95% CI: 68.1–74.1)	Recycle/return n = 149; 11.7% (95% CI: 9.4–14.5)	Have not gotten rid of coils/atomizers^a^ n = 194; 14.8% (95% CI: 12.4–17.6)
**Age Groups**	**Weighted % (95% CI)**	**Weighted % (95% CI)**	**Weighted % (95% CI)**	**Weighted % (95% CI)**	**Weighted % (95% CI)**	**Weighted % (95% CI)**	**Weighted % (95% CI)**
18-24	29.0 (25.9-32.3)	18.0 (14.3-22.4)	27.5 (23.1-32.4)	21.6† (8.9-43.5)	23.7 (21.0-26.6)	22.3 (15.8-30.5)	32.0 (23.8-41.5)
25-39	40.4 (36.9-44.1)	50.7 (44.3-57.2)	45.2 (39.7-50.8)	31.2† (12.3-59.4)	47.3 (43.4-51.3)	48.9 (38.3-59.5)	41.3 (30.7-52.7)
40-54	17.0 (14.1-20.4)	21.6 (15.7-28.8)	14.4 (10.9-18.7)	5.4† (1.1-21.9)	18.5 (15.2-22.2)	15.9 (9.0-26.6)	12.5 (7.7-19.7)
55+	13.6 (10.3-17.8)	9.7 (6.4-14.4)	13.0 (9.2-18.1)	41.9† (15.6-73.7)	10.5 (8.0-13.7)	13.0† (6.5-24.1)	14.2† (6.4-28.5)
**Sex**							
Male	56.0 (51.9-60.1)	67.1 (60.4-73.2)	47.9 (41.4-54.5)	23.7† (8.3-51.7)	55.7 (51.9-59.4)	65.8 (55.1-75.1)	51.7 (42.0-61.2)
Female	44.0 (39.9-48.1)	32.9 (26.8-39.6)	52.1 (45.5-58.6)	76.3† (48.3-91.7)	44.3 (40.6-48.1)	34.2 (24.9-44.9)	48.3 (38.8-58.0)
**Race/ethnicity**							
Non-Hispanic White	71.3 (66.9-75.4)	72.2 (65.2-78.3)	73.1 (67.6-77.9)	83.1† (63.7-93.2)	75.4 (71.8-78.7)	66.9 (56.0-76.2)	68.7 (59.3-76.7)
Non-Hispanic Black	8.1 (5.9-11.0)	8.4 (5.4-13.0)	8.0 (5.3-11.9)	0 (-,-)	7.1 (5.2-9.7)	9.7† (5.3-17.0)	9.0 (5.0-15.6)
Non-Hispanic Other/Multiple	6.3 (4.5-8.7)	5.7 (3.7-8.7)	8.1 (5.2-12.4)	6.7† (1.6-23.7)	6.4 (4.8-8.5)	7.6 (4.3-13.0)	7.1† (3.9-12.7)
Hispanic	14.2 (11.4-17.6)	13.6 (9.3-19.6)	10.8 (7.7-14.9)	10.3† (3.2-28.3)	11.1 (8.9-13.7)	15.8 (8.9-26.5)	15.2 (10.2-22.1)
**Education**							
Less than high school/some high school/no diploma	10.5 (8.0-13.6)	6.9 (4.3-10.9)	7.5 (4.6-12.0)	36.5† (11.7-71.4)	9.5 (7.4-12.1)	10.3† (5.5-18.3)	6.3† (3.1-12.2)
GED	6.5 (4.7-9.0)	6.4 (3.9-10.3)	7.3 (4.1-12.7)	0 (-,-)	8.5 (6.3-11.4)	5.4† (1.9-14.4)	5.3† (2.3-11.9)
High school graduate	27.9 (24.0-32.2)	31.2 (24.6-38.5)	24.3 (18.6-30.9)	2.9† (0.4-18.5)	28.5 (23.9-33.6)	30.1 (20.5-42.0)	31.3 (21.4-43.2)
Some college/2-year degree	40.7 (36.7-44.9)	40.6 (34.0-47.5)	42.6 (36.6-48.8)	50.0† (23.1-76.9)	40.3 (35.8-44.9)	40.3 (31.0-50.4)	42.2 (33.1-51.8)
College or more	14.3 (11.3-18.0)	14.9 (10.8-20.3)	18.3 (14.3-23.3)	10.6† (3.0-31.2)	13.2 (10.6-16.3)	13.9† (7.4-24.5)	14.9 (10.1-21.4)
**Income**							
<25,000	24.5 (20.6-28.9)	27.2 (21.1-34.2)	29.7 (24.1-35.9)	55.0† (27.2-80.0)	27.4 (24.1-31.0)	36.4 (28.1-45.7)	24.4 (17.6-32.8)
25,000-74,999	40.7 (36.0-45.6)	37.0 (31.8-42.5)	38.1 (32.8-43.6)	2.0† (0.2-17.0)	40.5 (36.8-44.2)	36.6 (27.6-46.6)	39.5 (29.5-50.5)
75,000+	29.8 (25.4-34.7)	32.5 (26.6-39.0)	26.7 (21.6-32.5)	34.3† (14.2-62.3)	27.5 (24.2-31.1)	22.9 (14.3-34.6)	25.7 (17.6-36.0)
Not reported	5.0 (3.5-6.9)	3.4 (1.9-5.8)	5.6 (3.5-8.9)	8.7† (2.5-26.3)	4.6 (3.3-6.3)	4.1† (1.6-10.0)	10.3 (6.2-16.5)
**Dual cigarette and ENP use**	47.0 (42.4-51.6)	40.9 (34.6-47.5)	42.5 (36.7-48.5)	58.0† (30.6-81.2)	43.9 (39.9-48.1)	34.6 (25.1-45.6)	52.4 (42.7-62.0)
**Frequency of use**							
Daily	55.5 (50.9-60.1)	57.5 (50.7-64.1)	50.2 (43.8-56.7)	32.8† (13.6-60.1)	64.8 (61.0-68.4)	51.9 (40.7-62.9)	34.0 (23.5-46.2)
Non-daily	44.5 (39.9-49.1)	42.5 (35.9-49.3)	49.8 (43.3-56.2)	67.2† (39.9-86.4)	35.2 (31.6-39.0)	48.1 (37.1-59.3)	66.0 (53.8-76.5)

Percentages are weighted using Wave 6 single-wave weights. Ns are unweighted. The total PATH Study Wave 6 sample includes 29,516 participants aged ≥18 years. Ns in this table may add to less than the totals due to missing data. CI, confidence interval; ENP, electronic nicotine products (commonly: e-cigarettes); PATH, Population Assessment of Tobacco and Health; landfill includes disposal in the trash; litter includes disposal on the ground; recycle/return includes returning it to a store, etc; other includes giving them to someone else. For batteries, litter disposal method N = 3; data are not presented in the table due to small n. For coils and atomizers, litter disposal method n = 8 and other disposal method N = 15; data are not presented in the table due to small n.

^†^Estimate should be interpreted with caution because it has low statistical precision. The estimate is based on a denominator sample size of less than 50, or the coefficient of variation of the estimate or its complement is larger than 30%.

^a^Adults that indicated that they used someone else’s product were combined with those who had not gotten rid of an empty battery/coil or atomizer.

#### Coils and atomizers.

Approximately 71.2% of adults usually discarded coils and atomizers in landfills (95% CI: 68.1, 74.1), and 11.7% usually recycled, returned, or reused them (95% CI: 9.4, 14.5). Demographic characteristics of those who used these two disposal methods were similar. However, 43.9% (95% CI: 39.9, 48.1) of those who usually disposed of their coils or atomizers in landfills used both ENP and cigarettes, and about two-thirds used ENP daily (64.8%; 95% CI: 61.0, 68.4). Of those who usually recycled their coils and atomizers, about a third dual used ENP and cigarettes (34.6%; 95% CI: 25.1, 45.6), and half used ENP daily (51.9%; 95% CI: 40.7, 62.9; Table 5).

#### Bottles or containers of E-liquid.

In response to questions about usual methods of disposal of empty bottles or containers of e-liquids, 70.9% (95% CI: 67.1, 74.5) of adults usually disposed of them in landfills, and 18.0% (95% CI: 15.1, 21.3) usually recycled, returned, or reused these components (see Table 6). The greatest share of adults was aged 25–39 (landfill: 44.6%; 95% CI: 40.4, 48.8; recycle: 50.5%; 95% CI: 41.7, 59.2), were males (landfill: 52.9%; 95% CI: 48.8, 56.8; recycle: 57.3%; 95% CI: 48.6, 65.6), and had some college education (landfill: 40.0%; 95% CI: 35.2, 45.1; recycle: 48.2%; 95% CI: 40.3, 56.1). Though similar proportions of adults used ENP daily between both disposal practices, fewer adults who usually recycled their empty bottles or e-liquid containers used both cigarettes and ENP (31.8%; 95% CI: 24.7, 39.8; see Table 6).

**Table 6:** Disposal of E-liquid Bottle and Leftover E-liquid Among Adults Who Currently Use ENP, Wave 6 (2021) of the PATH Study.

	Bottle or container of e-liquid (among users of refillable devices; n = 1,129)			Leftover or unused e-liquid (among users of refillable devices; n = 744)			
	Landfill n = 812; 70.9% (95% CI: 67.1–74.5)	Recycle/Return/Reuse n = 192; 18% (95% CI: 15.1–21.3)	Have not gotten rid of an empty one^a^ n = 119; 10.2% (95% CI: 8.4–12.3)	Landfill n = 369; 50.5% (95% CI: 45.8–55.2)	Litter/Sewer n = 92; 12.6% (95% CI: 9.7–16.2)	Have not gotten rid of leftover/unused e-liquid^a^ n = 240; 30.3% (95% CI: 26–34.9)	Gave it away/Other n = 32; 5.1%† (95% CI: 3.0–8.7)
**Age Groups**	**Weighted %** **(95% CI)**	**Weighted %** **(95% CI)**	**Weighted %** **(95% CI)**	**Weighted %** **(95% CI)**	**Weighted %** **(95% CI)**	**Weighted % (95% CI)**	**Weighted %** **(95% CI)**
18-24	24.7 (21.3-28.4)	19.8 (14.8-26.0)	29.9 (22.1-39.1)	23.9 (19.7-28.6)	22.6 (15.1-32.5)	27.7 (21.7-34.6)	14.0† (5.9-29.9)
25-39	44.6 (40.4-48.8)	50.5 (41.7-59.2)	39.9 (28.6-52.3)	48.5 (42.5-54.6)	46.1 (35.8-56.7)	43.1 (33.7-53.0)	31.8† (15.0-55.2)
40-54	19.0 (15.5-23.0)	16.0 (9.9-24.8)	12.5† (6.3-23.0)	18.8 (14.4-24.1)	16.3 (9.5-26.4)	20.8 (13.8-30.2)	17.3† (6.0-40.6)
55+	11.7 (8.8-15.5)	13.8 (8.6-21.2)	17.8† (9.2-31.5)	8.8 (5.6-13.5)	15.0† (8.1-26.1)	8.4 (4.7-14.4)	36.8† (13.7-68.1)
**Sex**							
Male	52.9 (48.8-56.8)	57.3 (48.6-65.6)	46.9 (34.7-59.5)	56.2 (49.9-62.4)	62.1 (49.7-73.1)	40.3 (33.8-47.2)	31.6† (13.3-58.2)
Female	47.1 (43.2-51.2)	42.7 (34.4-51.4)	53.1 (40.5-65.3)	43.8(37.6-50.1)	37.9 (26.9-50.3)	59.7 (52.8-66.2)	68.4† (41.8-86.7)
**Race/ethnicity**							
Non-Hispanic White	76.2 (72.3-79.7)	71.1 (62.1-78.7)	62.2 (50.9-72.4)	71.8 (65.7-77.2)	75.4 (63.6-84.3)	74.3 (68.2-79.5)	77.3† (57.3-89.6)
Non-Hispanic Black/other/Multiple	13.3 (10.7-16.4)	16.9 (10.9-25.4)	17.9 (10.6-28.7)	13.4 (9.9-17.9)	16.9 (9.3-28.7)	13.3 (9.2-19.0)	9.9† (3.5-24.8)
Hispanic	10.5 (7.9-13.7)	12.0 (7.5-18.7)	19.8 (13.1-28.9)	14.8 (10.5-20.3)	7.8† (3.8-15.1)	12.4 (8.8-17.2)	12.8† (4.7-30.7)
**Education**							
Less than high school/some high school/no diploma/GED	17.2 (13.8-21.2)	12.2 (7.5-19.2)	8.8† (3.7-19.6)	15.3 (11.0-20.8)	20.7† (11.1-35.2)	13.1 (8.9-18.7)	28.2† (9.4-59.7)
High school graduate	29.7 (24.5-35.5)	26.9 (19.8-35.4)	26.0 (16.6-38.2)	26.8 (21.0-33.6)	30.1 (20.0-42.7)	33.9 (26.2-42.6)	21.8† (8.8-44.7)
Some college/2-year degree	40.0 (35.2-45.1)	48.2 (40.3-56.1)	43.9 (33.4-55.0)	41.2 (34.9-47.8)	34.3 (23.5-47.0)	38.7 (31.2-46.9)	30.0† (13.7-53.5)
College or more	13.0 (10.2-16.6)	12.7 (8.6-18.3)	21.3 (12.7-33.5)	16.7 (12.0-22.8)	14.9† (8.1-25.9)	14.3 (9.8-20.3)	20.1† (5.5-52.0)
**Income**							
<25,000	28.8 (25.3-32.5)	23.3 (17.0-31.0)	26.1 (17.0-38.0)	27.2 (21.7-33.6)	33.9 (22.9-46.9)	27.1 (19.4-36.5)	34.2† (13.1-64.2)
25,000-74,999	42.4 (38.2-46.7)	36.9 (29.7-44.8)	37.2 (26.3-49.6)	42.0 (36.1-48.1)	36.9 (25.4-50.2)	41.3 (33.7-49.5)	21.7† (8.8-44.5)
75,000+	23.3 (19.4-27.8)	37.2 (28.8-46.5)	27.0 (17.4-39.4)	25.6 (20.6-31.2)	27.0 (15.5-42.7)	26.6 (20.5-33.7)	41.0† (19.9-66.0)
Not reported	5.6 (4.1-7.5)	2.5† (0.9-7.2)	9.6† (4.6-19.1)	5.2 (3.3-8.1)	2.2† (0.6-7.7)	5.0† (2.6-9.2)	3.1† (0.6-15.7)
**Dual cigarette and ENP use**	45.8 (41.6-50.2)	31.8 (24.7-39.8)	55.7 (44.7-66.2)	47.7 (41.5-54.0)	40.9 (28.0-55.3)	44.7 (36.3-53.5)	70.7† (47.7-86.5)
**Frequency of use**							
Daily	63.3 (59.2-67.2)	59.7 (49.0-69.5)	20.6 (13.1-30.9)	56.7 (50.2-63.0)	61.6 (48.9-72.9)	55.8 (46.3-64.9)	15.7† (6.4-33.6)
Non-daily	36.7 (32.8-40.8)	40.3 (30.5-51.0)	79.4 (69.1-86.9)	43.3 (37.0-49.8)	38.4 (27.1-51.1)	44.2 (35.1-53.7)	84.3† (66.4-93.6)

Percentages are weighted using Wave 6 single-wave weights. Ns are unweighted. The total PATH Study Wave 6 sample includes 29,516 participants aged ≥18 years. Ns in this table may add to less than the totals due to missing data. CI, confidence interval; ENP, electronic nicotine products (commonly: e-cigarettes); PATH, Population Assessment of Tobacco and Health; landfill includes disposal in the trash; litter includes disposal on the ground; sewer includes disposal in the sink or toilet; recycle/return/reuse includes returning it to a store, etc; other includes giving them to someone else. For bottles or containers of e-liquid, other disposal method n = 5; data are not presented in the table due to small n. For leftover e-liquid, recycle/return/reuse disposal method N = 11; data are not presented in the table due to small n. Estimates are collapsed due to small sample size (n = 1 or n = 2); for education, less than high school/some high school/no diploma was combined with GED; for ethnicity, non-Hispanic black was combined with non-Hispanic other race and multiple races.

^†^Estimate should be interpreted with caution because it has low statistical precision. The estimate is based on a denominator sample size of less than 50, or the coefficient of variation of the estimate or its complement is larger than 30%. *Column on littering for bottles or container for e-liquid is not presented due to small size (n < 3).

^a^Adults that indicated that they used someone else’s product were combined with those who had not gotten rid of an empty e-liquid bottle/leftover e-liquid.

#### Leftover or unused E-liquid.

Finally, adults were asked how they usually disposed of leftover or unused e-liquids. More than half (50.5%) of them usually disposed of them in landfills (95% CI: 45.8, 55.2), 12.6% (95% CI: 9.7, 16.2) usually disposed of leftover or unused e-liquids as litter or in sewage, and 30.3% (95% CI: 26.0–34.9) had not gotten rid of unused e-liquid. Demographic characteristics for those disposing of leftover or unused e-liquids were similar. The largest share were adults aged 25–39 (landfill: 48.5%; 95% CI: 42.5, 54.6; litter or sewage: 46.1%; 95% CI: 35.8, 56.7), predominantly male (landfill: 56.2%; 95% CI: 49.9, 62.4, litter or sewage: 62.1%; 95% CI: 49.7, 73.1), and had some college education (landfill: 41.2%; 95% CI: 34.9, 47.8; litter or sewage: 34.3%; 95% CI: 23.5, 47.0). Among those who usually disposed of liquids in landfills, almost half dual used ENP and cigarettes (47.7%; 95% CI: 41.5, 54.0) and more than half used ENP daily (56.7%; 95% CI: 50.2, 63.0; see Table 6).

### Sensitivity analysis for mode differences

In Wave 6 (2021), due to the continued COVID-19 pandemic, respondents were allowed to complete the interview by telephone if they did not want an in-person interview (as was done in Waves 1–5). This may have impacted our results as respondents completing an interview over the phone may be susceptible to more socially desirable responding with respect to their disposal practice (for example underreporting littering) than when completing the interview on a laptop during in-person visits. We conducted a sensitivity analysis to determine if interview mode impacted our conclusion by stratifying the analysis by interview type (telephone or in-person).

Among adults who currently smoked cigarettes and were interviewed over telephone, 89.4% (95% CI: 88.0, 90.7) usually disposed of their cigarette butts in landfills and 8.3% (95% CI: 7.1, 9.6) usually disposed of butts as litter or in sewage. Among adults who were interviewed in-person, 90.0% (95% CI: 88.4, 91.3) and 8.2% (95% CI: 7.0, 9.6) usually disposed of their cigarette butts in landfills or littered respectively. Among adults who used ENP, the disposal practices were similar for telephone and in-person modes (telephone interview: 84.6%; 95% CI: 81.2, 87.5 versus in-person interview: 81.7%; 95% CI: 77.4, 85.3% usually discarded disposable devices in landfill; telephone interview: 4.4%; 95% CI: 3.0, 6.4 compared to in-person: 7.6%; 95% CI: 5.2, 10.8 usually recycled, returned, or reused their disposable ENP). We found that the overall pattern of results is consistent with our findings and the mode of data collection did not change our conclusions.

## Discussion

We found that only 8.3% of adults who currently smoke cigarettes littered their cigarette butts into the environment. This estimate is much lower than results from previous studies. For example, Smith and Novotny found littering rates between 45%−75% based on industry-reported data [38]. Self-reported studies showed cigarette butt littering by almost 50% of respondents, while observational studies of people who smoke cigarettes reported much higher rates [39–42]. The differences between observational and self-report data suggest that people who smoke cigarettes may underestimate or underreport their butt littering, which is consistent with research that has found differences in self-reported and objective measures of environmental behaviors [40,43,44]. In any case, the low rates reported here could be due to many reasons, one of which could be how the PATH Study interview asks people who smoke where they “usually” throw away their cigarette butts and does not capture the actual practice of disposal, which could reinforce some biases such as social desirability and recall bias of past behavior [43,45]. Even though disposing of cigarette butts in an ashtray or trash can (i.e., landfill) was the most common practice in our study, it is possible that those who usually disposed of cigarette butts in landfills still littered when they could not utilize a trash receptacle.

Despite the growing environmental concern regarding ENP disposal, very little is known about the environmental impacts of these practices. ENP waste is generated from non-biodegradable and poorly recyclable plastic (including pods, device bodies, cartridges), electronics (circuit boards, batteries), and hazardous chemicals such as nicotine [11,13], PAHs [11,13], pesticides [46], tar [47], and heavy metals [6,11,13,48]. Moreover, heavy metals may be released if ENP are disposed of in landfills [6]. Our findings show that the most common disposal method for ENP waste including empty pods or cartridges, batteries, coils or atomizers, and bottles or containers of e-liquid was disposing of them in a landfill (resulting from throwing in trash). For disposable ENP, reliable manufacturers’ recycling package instructions as well as data on actual recycling are largely unavailable [49]. This is concerning because the use of disposable ENP has grown drastically in recent years. Given the growing of single-use ENP that are largely non-biodegradable, poorly recyclable, and contain both electronic and hazardous waste, there is a need to understand more about the potential environment consequences of ENP waste, whether disposed of in landfills or into the environment [48].

Recycling of batteries, while still low, was more common than other ENP components. Disposal of batteries in landfills is of concern because batteries can be explosive, are a fire risk at both waste and recycling facilities, and as batteries degrade, their toxic compounds may leach out of landfills into the environment [50,51]. Furthermore, it is also to be noted that recycling of batteries from ENPs is a growing challenge, including that the design feature of several devices makes it difficult for consumers to remove them. This is especially true for disposable ENPs, which have sealed units with batteries integrated into the device with no easy way of removing them. This could be a potential limitation in implementing best disposal practices, such as recycling.

One important finding from our study is the disposal practice for left over or unused e-liquid. About 50% of respondents disposed this waste into landfill, while 12.6% littered leftover or unused e-liquid into the environment. The U.S. EPA lists nicotine as an acute hazardous waste; this requires that certain commercial producers not to discard nicotine-containing e-liquid waste into regular waste or pour down sinks. However, while the Resource Conservation and Recovery Act (RCRA) classifies nicotine as a hazardous substance, this act does not regulate the disposal of ENP by consumers [49]. These nicotine-containing items should be taken safely to a hazardous waste facility, and the PATH Study did not inquire about this practice.

Safe disposal guidance for cigarette butts and ENP components is limited and not widely known, and thus educating those who use cigarettes and ENPs on the environmental impact of disposal practices is important. Recycling ENPs can be challenging due to their mix of materials, including batteries, plastics, and hazardous substances. Available disposal and recycling for ENPs include e-waste recycling facilities which handle lithium batteries and circuit boards, household hazardous waste facilities that are offered by local agencies that ensure safe disposal of toxic components, mail-back recycling services that are offered by some organizations and manufacturers, retail take-back programs that offer collection bins or return programs for used devices, pods, and batteries. For proper cigarette butt disposal using designated cigarette receptables such as bins or ashtrays, participating in cigarette butt recycling initiatives, placing butts in general waste bins and avoiding littering in drains and waterways are some ways of properly disposing cigarette butts. Our findings can be valuable in understanding the magnitude of waste from cigarette butts and ENPs and may help identify knowledge gaps in current disposal policies.

Limitations of the study include recall and self-reporting biases. As the interview asked adults where they “usually” throw away their cigarette butts and ENP components, it did not assess actual practice. This could have introduced some social desirability biases, especially around littering, which may have been underreported, while practices such as recycling, reusing, and returning, which are more environmentally friendly, may have been overreported. It is also not known what ultimately happened to the cigarette butts; butts that were disposed of in ashtrays could have been littered as opposed to ending up in landfills. In addition, it could not be determined if other methods of disposal, such as recycling, eventually resulted in landfill disposition, especially given the limited recycling options for products like disposable ENPs. These facets of the interview design may contribute, in part, to some observed differences in disposal practices in our findings compared to previous studies, both in terms of overall rates of littering and differences by demographic variables [40,52,53]. Although the PATH Study interview asks detailed questions about tobacco use, it did not explicitly ask about perceptions and attitudes toward disposal that would inform intervention efforts to create awareness of safe disposal practices. Data for the current analysis are cross-sectional, and thus causal relationships or inferences about how practices in disposal may change over time among those who smoke cigarettes or use ENP cannot be assumed from the findings. However, this study has several strengths, such as the first nationally representative self-reported data on tobacco product littering, and the first analysis of data on roll-your-own tobacco pouches, and ENP components disposal practices.

## Conclusions

While our study showed low rates of littering cigarette butts as a usual disposal practice, the results suggest that even these relatively low rates may produce a significant volume of littered cigarette butts (9.2 billion in 2021). Our estimate is conservative, given the possibility of self-report biases and how the questions were asked, as previously discussed. Other estimates of cigarette butts littered have been reported, but these estimates do not take into account the number of cigarettes actually consumed [53–57]. Rather than using cigarette smoking prevalence and reported number of cigarettes smoked per day to estimate total cigarette consumption [56], United States Cigarette sales data, such as from the NielsenIQ Retail Scanner Data [57], could be used to estimate the total number of cigarette butts that could possibly be disposed [58]. Whether using manufactured cigarette totals or cigarette sales data, there is insufficient corresponding information on the actual number of these cigarettes that are littered as butts. Nevertheless, this PATH Study provides a nationally representative estimate of littered cigarette butts in the U.S. given that participants report both the number of cigarettes they smoked and how often they littered their cigarette butts.

With billions of cigarette butts disposed of improperly in the U.S., comprehensive efforts to improve tobacco product waste management are needed. Our findings may inform efforts by environmental organizations, state and local governments, and industry to better inform consumers about how to dispose of cigarette butts and ENP waste safely and to take other effective policy actions [59]. These may include efforts to limit outdoor smoking and ENP use and to address hazardous and non-biodegradable tobacco product ingredients and components. Improper disposal of cigarette butts and ENP is a rising global environmental concern that should be addressed to avoid environmental consequences. While these data are specific to the U.S., they may help inform both the U.S. and other countries about practices and challenges in disposing of tobacco product waste, particularly multi-component products like ENPs that have surged in popularity, and ultimately aid stakeholders in their local education, policy, or research efforts.

## Supporting information

S1 Table Other-specify response recodes for cigarette butt disposal practices, Wave 6 (2021) of the PATH Study. (DOCX)

S2 Table Other-specify response recodes for disposable electronic nicotine product disposal practices, Wave 6 (2021) of the PATH Study. (DOCX)

S3 Table Other-specify response recodes for empty pod or cartridge disposal practices, Wave 6 (2021) of the PATH Study. (DOCX)

S4 Table Other-specify response recodes for battery disposal practices, Wave 6 (2021) of the PATH Study. (DOCX)

S5 Table Other-specify response recodes for coil or atomizer disposal practices, Wave 6 (2021) of the PATH Study. (DOCX)

S6 Table Other-specify response recodes for empty bottle or container of e-liquid disposal practices, Wave 6 (2021) of the PATH Study. (DOCX)

S7 Table Other-specify response recodes for leftover e-liquid disposal practices, Wave 6 (2021) of the PATH Study. (DOCX)

S8 Appendix A Preferred Reporting Items for Complex Sample Survey Analysis (PRICSSA) checklist for the PATH Study. (DOCX)

S9 Appendix B Disposal measures for cigarettes and ENP components in PATH Study questionnaire at Wave 6. (DOCX)
