# Correction: Disposal practices of cigarettes and electronic nicotine products among adults, findings from Wave 6 (2021) of the PATH Study

**DOI:** 10.1371/journal.pone.0344579

**Published:** 2026-03-06

**Authors:** Eva Sharma, Johanna Dubsky, Katherine V. Garcia-Rosales, Michael Halenar, Adam F. Benson, Alexander Maki, Andrea L. Ruybal, Aura Lee Morse, Michael D. Sawdey, Rudaina Alrefai-Kirkpatrick, Silvana Skara, Susana Addo Ntim, Hoshing W. Chang, Ther W. Aung, Heather L. Kimmel, Jennifer L. Pearson, Maansi Bansal-Travers, Kristie A. Taylor, Andrew Hyland, Thomas E. Novotny

The Data Availability statement for this article is incorrect. The correct statement is: Data are available in a public, open access repository. The data underlying this article are available through the United States Department of Health and Human Services. National Institutes of Health, National Institute on Drug Abuse, and United States Department of Health and Human Services. Food and Drug Administration. Center for Tobacco Products. Population Assessment of Tobacco and Health (PATH) Study [United States] Restricted-Use Files. Inter-university Consortium for Political and Social Research [distributor], 2021-06-29. The PATH Study data are available via download or application at https://doi.org/10.3886/Series606.
