# Supplementary material for: Disposal practices of cigarettes and electronic nicotine products among adults, findings from Wave 6 (2021) of the PATH Study
Source: PLoS One. 2025 Dec 9;20(12):e0338007. doi: 10.1371/journal.pone.0338007 (PMC12688147; doi:10.1371/journal.pone.0338007)
Supplement: S1 Table — (DOCX) [file pone.0338007.s001.docx]

| **Supplemental (S)1 Table. Other-specify response recodes for cigarette butt disposal practices, Wave 6 (2021) of the PATH Study** | | | | | |
| --- | --- | --- | --- | --- | --- |
| **R06_AC8800_OS: Where you usually throw away cigarette butts after you finish smoking a cigarette: Somewhere else - specify** | **Landfill** | **Litter/Sewer** | **Container** | **Other** | **System Missing** |
| -8 |  |  |  | X |  |
| -7 |  |  |  |  | X |
| 5 GALLON BUCKET OUTSIDE OF HOUSE |  |  | X |  |  |
| A MONSTER CAN FILLED WITH WATER THAT HAS A SCREW ON CAP |  |  | X |  |  |
| ALUMINUM CAN |  |  | X |  |  |
| AN EMPTY SODA CAN OR BOTTLE WITH WATER IN IT |  |  | X |  |  |
| ANYWHERE I CAN |  |  |  | X |  |
| ANYWHERE WHILE AT MY FRIEND'S HOUSE |  |  |  | X |  |
| BACK IN THE PACK |  |  |  | X |  |
| BACK POCKET |  |  |  | X |  |
| BEER CAN |  |  | X |  |  |
| BOTTLE |  |  | X |  |  |
| BOTTLE OF WATER |  |  | X |  |  |
| BOTTLE OF WATER WITH A LID ON IT |  |  | X |  |  |
| BOX |  |  | X |  |  |
| BUCKET |  |  | X |  |  |
| BUCKET OF WATER OUTSIDE THE HOUSE |  |  | X |  |  |
| BUCKET SAND |  |  | X |  |  |
| BUTT CAN |  |  | X |  |  |
| CAN |  |  | X |  |  |
| CAN ON FRONT PORCH |  |  | X |  |  |
| CAN OUTSIDE |  |  | X |  |  |
| CAN OUTSIDE BECAUSE I ONLY SMOKE OUTSIDE MY HOME |  |  | X |  |  |
| CLOSED CONTAINER |  |  | X |  |  |
| COFFEE CAN |  |  | X |  |  |
| COFFEE GROUND BOX |  |  | X |  |  |
| DISPOSABLE BOTTLE WITH WATER |  |  | X |  |  |
| DO NOT SMOKE |  |  |  |  | X |
| DO NOT SMOKE CIGARETTES - USE VAPE PEN AND THROW IN TRASH |  |  |  |  | X |
| DOES NOT APPLY. ONLY SMOKE VAPE PENS. |  |  |  |  | X |
| DON'T |  |  |  |  | X |
| DON'T SMOKE |  |  |  |  | X |
| EMPTY CAN |  |  | X |  |  |
| EMPTY FLOWER POT OUTSIDE |  |  | X |  |  |
| EMPTY PLASTIC BOTTLE |  |  | X |  |  |
| EMPTY PLASTIC GALLON JUG |  |  | X |  |  |
| EMPTY POP BOTTLE |  |  | X |  |  |
| EMPTY POP CAN |  |  | X |  |  |
| EN UN ENVASE CON AGUA |  |  | X |  |  |
| EN UNA CANTARA QUE ESTA AFUERA |  |  | X |  |  |
| EN UNA LATA DE UNA SODA O ALGO |  |  | X |  |  |
| FIRE PIT |  |  |  | X |  |
| HAVE ONLY TAKEN A PUFF FROM SOMEONE ELSE'S CIGARETTE. |  |  |  | X |  |
| I DO NOT SMOKE CIGARETTES QUESTIONS ARE IRRELEVANT GREAT SURVEY |  |  |  |  | X |
| I DON'T SMOKE |  |  |  |  | X |
| I HAVE A BUCKET AT HOME MADE FOR THEM. THAT MY CHILDREN CAN ACCESS. |  |  | X |  |  |
| I HAVE NOT HAD A CIGARETTE SINCE I WAS A TEEN |  |  |  |  | X |
| I'VE NEVER SMOKED A WHOLE CIGARETTE IN MY LIFE - I GIVE IT BACK TO THE PERSON |  |  |  | X |  |
| KEEPS IN A SMALL BAG TO REUSE |  |  |  | X |  |
| LANDSCAPE (BUSHES) |  | X |  |  |  |
| MY POCKET |  |  |  | X |  |
| MY PURSE |  |  |  | X |  |
| NEVER |  |  |  |  | X |
| NEVER FINISHED ONE...1 OR 2 PUFFS AND GIVE BACK TO PERSON THAT I ASKED FOR A DRAG, ONLY HAVE DONE THIS 3 OR 4 TIMES EVER |  |  |  | X |  |
| ONLY SMOKE A FEW PUFFS SO GIVES THE CIGARETTE BACK TO THE OWNER |  |  |  | X |  |
| ONLY SMOKE EVERY 6 MONTHS SO I DON'T TRACK OF IT |  |  |  |  | X |
| OUT OF THE WINDOW |  | X |  |  |  |
| OUTSIDE IN THE GARAGE |  |  |  | X |  |
| PLASTIC BAG UNTIL IT'S FULL |  |  | X |  |  |
| PLASTIC WATER FILLED CONTAINER |  |  | X |  |  |
| POUCH CARRIES AND FILLS IT |  |  |  | X |  |
| RECYCLE BIN |  |  | X |  |  |
| SMALL FLOWER OUTSIDE HOME |  |  | X |  |  |
| SODA CAN |  |  | X |  |  |
| SOMEWHERE ELSE. |  |  |  | X |  |
| TIN CAN |  |  | X |  |  |
| TRAY |  |  | X |  |  |
| VASE |  |  | X |  |  |
| WHERE I'M AT |  |  |  | X |  |
| WHEREVER IS MOST CONVENIENT FROM THE LIST |  |  |  | X |  |
| WOOD BURNER |  |  |  | X |  |
