# Supplementary material for: Disposal practices of cigarettes and electronic nicotine products among adults, findings from Wave 6 (2021) of the PATH Study
Source: PLoS One. 2025 Dec 9;20(12):e0338007. doi: 10.1371/journal.pone.0338007 (PMC12688147; doi:10.1371/journal.pone.0338007)
Supplement: S2 Table — (DOCX) [file pone.0338007.s002.docx]

| **S2 Table.** **Other-specify response recodes for disposable electronic nicotine product disposal practices, Wave 6 (2021) of the PATH Study** | | | | | | |
| --- | --- | --- | --- | --- | --- | --- |
| **R06_AV8810_OS: What you usually do with your disposable electronic nicotine product when it is empty: Something else - specify** | **Landfill** | **Litter** | **Recycle/return/reuse** | **Have not gotten rid of an empty one** | **Other** | **System Missing** |
| -8 |  |  |  |  | X |  |
| BECAUSE IT WAS NOT MINE |  |  |  | X |  |  |
| GAVE THEM AWAY |  |  |  |  | X |  |
| GIVE IT BACK TO MY FRIEND |  |  |  | X |  |  |
| GIVE IT BACK TO THE PERSON WHO LET ME USE IT |  |  |  | X |  |  |
| GIVE IT TO FRIEND |  |  |  |  | X |  |
| GIVE IT TO MY BROTHER TO RECHARGE IT FOR ME |  |  |  |  |  | X |
| HAVE NEVER OWNED A NICOTINE PRODUCT |  |  |  |  |  | X |
| I DO NOT BUY MY OWN ECIGARETTES, BUT OCCASIONALLY I WILL TAKE A PUFF FROM A FRIEND'S. |  |  |  | X |  |  |
| I DON'T |  |  |  |  |  | X |
| I HAVE NEVER EMPTIED ONE, IT'S LIKE PUFF PUFF, GIVE BACK |  |  |  | X |  |  |
| I USE SOMEONE ELSE'S, NEVER HAVE MY OWN |  |  |  | X |  |  |
| IT WAS MY FRIEND'S. I JUST TRIED IT ONCE |  |  |  | X |  |  |
| LOSE IT |  |  |  |  |  | X |
| NEVER OWNED ONE. JUST TAKEN PUFFS OFF OF FRIENDS' |  |  |  | X |  |  |
| RE-PACK THEM |  |  | X |  |  |  |
| RETURN IT TO THE OWNER |  |  |  | X |  |  |
| SOMEONE ELSE GETS RID OF IT |  |  |  | X |  |  |
| THEY ARE NOT MINE SO I DO NOT DISPOSE OF THEM |  |  |  | X |  |  |
