# Supplementary material for: Disposal practices of cigarettes and electronic nicotine products among adults, findings from Wave 6 (2021) of the PATH Study
Source: PLoS One. 2025 Dec 9;20(12):e0338007. doi: 10.1371/journal.pone.0338007 (PMC12688147; doi:10.1371/journal.pone.0338007)
Supplement: S3 Table — (DOCX) [file pone.0338007.s003.docx]

| **S3 Table.** **Other-specify response recodes for empty pod or cartridge disposal practices, Wave 6 (2021) of the PATH Study** | | | | | | |
| --- | --- | --- | --- | --- | --- | --- |
| **R06_AV8811_OS: What you usually do with a pod or cartridge when it is empty: Something else - specify** | **Landfill** | **Litter** | **Recycle/return/reuse** | **Have not gotten rid of an empty one** | **Other** | **System Missing** |
| DON'T |  |  |  |  |  | X |
| GAVE IT TO SOMEONE ELSE BECAUSE I DIDN'T FINISH IT |  |  |  |  | X |  |
| GIVE IT BACK TO SOMEONE ELSE |  |  |  | X |  |  |
| I LOSE THEM |  |  |  |  |  | X |
| IT IS SOMEONE ELSE'S |  |  |  | X |  |  |
