# Supplementary material for: Disposal practices of cigarettes and electronic nicotine products among adults, findings from Wave 6 (2021) of the PATH Study
Source: PLoS One. 2025 Dec 9;20(12):e0338007. doi: 10.1371/journal.pone.0338007 (PMC12688147; doi:10.1371/journal.pone.0338007)
Supplement: S4 Table — (DOCX) [file pone.0338007.s004.docx]

| **S4 Table.** **Other-specify response recodes for battery disposal practices, Wave 6 (2021) of the PATH Study** | | | | | | |
| --- | --- | --- | --- | --- | --- | --- |
| **R06_AV8812_OS: What you usually do with the battery for your electronic nicotine product after it no longer works or is needed: Something else - specify** | **Landfill** | **Litter** | **Recycle/return** | **Have not gotten rid of an empty one** | **Other** | **System Missing** |
| -8 |  |  |  |  | X |  |
| BUYS RECHARGEABLE BATTERY |  |  |  |  |  | X |
| CHARGE IT |  |  |  |  |  | X |
| CHARGE IT (RECHARGEABLE BATTERY) |  |  |  |  |  | X |
| CHARGED THEM |  |  |  |  |  | X |
| CHARGES EXTERNALLY DON'T USE BATTERY |  |  |  |  |  | X |
| DISPOSE IT PROPERLY |  |  |  |  | X |  |
| DOES NOT HAVE A BATTERY |  |  |  |  |  | X |
| DOES NOT HAVE A BATTERY - CHARGES ON PHONE |  |  |  |  |  | X |
| DON'T OWN ANY E-BATTERIES |  |  |  |  |  | X |
| GIVE BACK TO OWNER |  |  |  | X |  |  |
| GIVE IT AWAY |  |  |  |  | X |  |
| GIVE IT AWAY OR THROW IT AWAY |  |  |  |  | X |  |
| GIVE IT BACK TO THE OWNER |  |  |  | X |  |  |
| GIVE IT TO A FRIEND |  |  |  |  | X |  |
| GIVE IT TO SOMEONE ELSE |  |  |  |  | X |  |
| HAS A CHARGER |  |  |  |  |  | X |
| I CHARGE THEM AND KEEPING USING UNTIL I LOSE IT |  |  |  |  |  | X |
| I DON'T OWN AN ELECTRONIC NICOTINE PRODUCT |  |  |  |  |  | X |
| I DON'T OWN ONE |  |  |  |  |  | X |
| I JUST HAVE TASTE IT, I HAVE NOT BUY MY OWN. |  |  |  | X |  |  |
| I JUST RECHARGE THE BATTERIES TO USE AGAIN. |  |  |  |  |  | X |
| I THINK A FRIEND RECHARGES IT. |  |  |  |  |  | X |
| I TRY TO SEE IF I CAN CHARGE IT |  |  |  |  |  | X |
| I USUALLY GIVE IT AWAY |  |  |  |  | X |  |
| IT DOES NOT HAVE A BATTERY |  |  |  |  |  | X |
| IT DOES NOT HAVE A BATTERY. |  |  |  |  |  | X |
| IT IS NOT MINE SOMEONE GIVES IT TO ME |  |  |  | X |  |  |
| LOST IT, CAN'T FIND IT. |  |  |  |  |  | X |
| NEVER BOUGHT ONE |  |  |  |  |  | X |
| NOTHING; IT IS AN INTERNAL BATTERY |  |  |  |  |  | X |
| ONLY TRIED AN ELECTRONIC NICOTINE PRODUCT ONCE IN THE PAST MONTH. |  |  |  |  |  | X |
| RECHARGE IT |  |  |  |  |  | X |
| RECHARGEABLE |  |  |  |  |  | X |
| REUSABLE |  |  |  |  |  | X |
| SOMETHING ELSE |  |  |  |  | X |  |
| THE OTHER PERSON TAKES CARE OF THAT |  |  |  | X |  |  |
| USE UNA SOLA VEZ |  |  |  |  |  | X |
| WHAT BATTERY? |  |  |  |  |  | X |
