# Supplementary material for: Disposal practices of cigarettes and electronic nicotine products among adults, findings from Wave 6 (2021) of the PATH Study
Source: PLoS One. 2025 Dec 9;20(12):e0338007. doi: 10.1371/journal.pone.0338007 (PMC12688147; doi:10.1371/journal.pone.0338007)
Supplement: S5 Table — (DOCX) [file pone.0338007.s005.docx]

| **S5 Table.** **Other-specify response recodes for coil or atomizer disposal practices, Wave 6 (2021) of the PATH Study** | | | | | | |
| --- | --- | --- | --- | --- | --- | --- |
| **R06_AV8814_OS: What you usually do with the coils or atomizers for your electronic nicotine product after they no longer work: Something else - specify** | **Landfill** | **Litter** | **Recycle/return/reuse** | **Have not gotten rid of an empty one** | **Other** | **System Missing** |
| -8 |  |  |  |  | X |  |
| CHARGE IT |  |  |  |  |  | X |
| DON'T OWN ANY |  |  |  |  |  | X |
| GIVE BACK TO OWNER |  |  |  | X |  |  |
| GIVE IT BACK TO THE OWNER |  |  |  | X |  |  |
| GIVE IT TO MY FRIEND |  |  |  |  | X |  |
| GIVE THEM TO SPOUSE |  |  |  |  | X |  |
| I DON'T OWN ONE |  |  |  |  |  | X |
| I DON'T USE THEM |  |  |  |  |  | X |
| I HAVE NO IDEA WHAT THOSE THINGS ARE. |  |  |  |  |  | X |
| I NEVER HAD ANY VAPOR OR ELECTRONIC CIGARETTES IN MY POSSESSION. |  |  |  |  |  | X |
| IT IS NOT MINE |  |  |  | X |  |  |
| ONLY TRIED AN ELECTRONIC NICOTINE PRODUCT ONCE IN THE PAST MONTH. |  |  |  |  |  | X |
| SOLO LO USE UNA SOLA VEZ |  |  |  |  |  | X |
| THE OTHER PERSON TAKES CARE OF THAT |  |  |  | X |  |  |
