# Supplementary material for: Disposal practices of cigarettes and electronic nicotine products among adults, findings from Wave 6 (2021) of the PATH Study
Source: PLoS One. 2025 Dec 9;20(12):e0338007. doi: 10.1371/journal.pone.0338007 (PMC12688147; doi:10.1371/journal.pone.0338007)
Supplement: S6 Table — (DOCX) [file pone.0338007.s006.docx]

| **S6 Table.** **Other-specify response recodes for empty bottle or container of e-liquid disposal practices, Wave 6 (2021) of the PATH Study** | | | | | | |
| --- | --- | --- | --- | --- | --- | --- |
| **R06_AV8816_OS: What you usually do with a bottle or container of e-liquid when it is empty: Something else - specify** | **Landfill** | **Litter** | **Recycle/return /reuse** | **Have not gotten rid of an empty one** | **Other** | **System Missing** |
| GIVE BACK TO OWNER |  |  |  | X |  |  |
| GIVE IT BACK TO THE OWNER |  |  |  | X |  |  |
| GIVE IT TO MY FRIEND |  |  |  |  | X |  |
| I DON'T OWN ONE |  |  |  |  |  | X |
| I HAVE NEVER THROWN AWAY BECAUSE I JUST USE WITH MY FRIEND AT HIS HOUSE. THIS BELONGS TO HIM. |  |  |  | X |  |  |
| I USUALLY RE-USE IT |  |  | X |  |  |  |
| N/A |  |  |  |  |  | X |
| ONLY TRIED SOMEONE ELSE'S PRODUCT ONCE. DID NOT DISPOSE OF IT. |  |  |  | X |  |  |
| PRODUCT WAS NOT MINE I JUST TRIED IT THE ONE TIME |  |  |  | X |  |  |
| REFILL |  |  | X |  |  |  |
| SOLO LO USE UNA SOLA VEZ |  |  |  |  |  | X |
| THE OTHER PERSON TAKES CARE OF THAT |  |  |  | X |  |  |
