# Supplementary material for: Disposal practices of cigarettes and electronic nicotine products among adults, findings from Wave 6 (2021) of the PATH Study
Source: PLoS One. 2025 Dec 9;20(12):e0338007. doi: 10.1371/journal.pone.0338007 (PMC12688147; doi:10.1371/journal.pone.0338007)
Supplement: S7 Table — (DOCX) [file pone.0338007.s007.docx]

| **S7 Table.** **Other-specify response recodes for leftover e-liquid disposal practices, Wave 6 (2021) of the PATH Study** | | | | | | | |
| --- | --- | --- | --- | --- | --- | --- | --- |
| **R06_AV8815_OS: What you usually do with leftover or unused e-liquid: Something else - specify** | **Landfill** | **Litter** | **Recycle/return/reuse** | **Have not gotten rid of an empty one** | **Gave it away** | **Other** | **System Missing** |
| ASK A FRIEND IF THEY WANT IT |  |  |  |  |  | X |  |
| DID NOT OWN THE PRODUCT. SOMEONE ELSE DISPOSED OF IT. |  |  |  | X |  |  |  |
| GAVE IT TO ANOTHER PERSON |  |  |  |  | X |  |  |
| GAVE IT TO FRIENDS |  |  |  |  | X |  |  |
| GIVE IT AWAY |  |  |  |  | X |  |  |
| GIVE IT AWAY TO SOMEONE ELSE |  |  |  |  | X |  |  |
| GIVE IT AWAY TO SOMEONE WHO WILL USE IT |  |  |  |  | X |  |  |
| GIVE IT BACK TO OWNER |  |  |  | X |  |  |  |
| GIVE IT TO A FRIEND |  |  |  |  | X |  |  |
| GIVE IT TO MY DAUGHTER |  |  |  |  | X |  |  |
| GIVE IT TO SOMEONE |  |  |  |  | X |  |  |
| GIVE IT TO SOMEONE ELSE |  |  |  |  | X |  |  |
| GIVE IT TO SOMEONE ELSE WHO CAN USE IT |  |  |  |  | X |  |  |
| HOLD ON TO IT, AND GIVE IT TO SOMEONE ELSE TO USE |  |  |  |  | X |  |  |
| I DON'T OWN ONE |  |  |  |  |  |  | X |
| I LET IT DRY OUT TO EMPTY IT AND FILL IT BACK UP. |  |  | X |  |  |  |  |
| I MIX THEM UP IN THE NEW BOTTLE |  |  | X |  |  |  |  |
| MIX IT WITH A DIFFERENT FLAVOUR |  |  | X |  |  |  |  |
| MIX WITH DIFFERENT FLAVOR |  |  | X |  |  |  |  |
| N/A |  |  |  |  |  |  | X |
| RECYCLE IT |  |  | X |  |  |  |  |
| SAVE IT FOR ANOTHER PERSON |  |  |  |  |  | X |  |
| SOLO LO USE UNA SOLA VEZ |  |  |  |  |  |  | X |
| THE OTHER PERSON TAKES CARE OF THAT |  |  |  | X |  |  |  |
| USE IT |  |  |  |  |  |  | X |
