# Supplementary material for: Disposal practices of cigarettes and electronic nicotine products among adults, findings from Wave 6 (2021) of the PATH Study
Source: PLoS One. 2025 Dec 9;20(12):e0338007. doi: 10.1371/journal.pone.0338007 (PMC12688147; doi:10.1371/journal.pone.0338007)
Supplement: S8 Appendix A — (DOCX) [file pone.0338007.s008.docx]

**Appendix A: Preferred Reporting Items for Complex Sample Survey Analysis (PRICSSA) Checklist for the PATH Study**

| **PRICSSA Item** | **PATH Study (Wave 6; 2021)** |
| --- | --- |
| Data collection mode(s) | In-person interviews using the audio computer-assisted self-interviewing (ACASI) and telephone interview using computer-assisted telephone interviewing (CATI) |
| Dates of data collection | March 1, 2021 -November 30, 2021 |
| Target population | U.S. Civilian, non-institutionalized population aged 18years and older in the United States. |
| Populations excluded | Individuals excludes include those who are institutionalized setting (e.g., prisons or long-term care facilities), active-duty military, and non-United States residents. |
| Sample Design | The PATH Study recruitment employed a stratified address-based, area-probability sampling design that oversampled adults who use tobacco, young adults (aged 18-24), and African American adults. |
| Variance estimation | Balanced repeated replication (BRR) method |
| Weight and design variables | Weight variables: Full sample and replicate weights  Design variables: Primary sampling unit (PSU) and stratum |
| Final sample weight | R06_A_S04WGT |
| Replicate weights/Stratum | R06_A_S04WGT1-R06_A_S04WGT100 |
| Unweighted total sample size | 29,516 |
| Weighted total sample size (estimate) | 248,191,625 |
| Response rate | 73.5% |
| Location of example code | PATH Study Restricted Use Files User Guide at <https://doi.org/10.3886/Series606> |
