# Supplementary material for: Disposal practices of cigarettes and electronic nicotine products among adults, findings from Wave 6 (2021) of the PATH Study
Source: PLoS One. 2025 Dec 9;20(12):e0338007. doi: 10.1371/journal.pone.0338007 (PMC12688147; doi:10.1371/journal.pone.0338007)
Supplement: S9 Appendix B — (DOCX) [file pone.0338007.s009.docx]

**APPENDIX B: Disposal measures for cigarettes and ENP components in PATH Study questionnaire at Wave 6**

| **Variable definition** | **Question** | **Who is asked** | **Questionnaire response options** | **Recoded response options** |
| --- | --- | --- | --- | --- |
| Where respondent disposes of cigarette butts | After you finish smoking a cigarette, where do you usually throw away cigarette butts? | Adult respondents who currently smoke manufactured cigarettes. | 1) In an ash tray or cigarette disposal, 2) In the trash, 3) On the ground, 4) In the sink or toilet, 5) Out of a car window, 6) Somewhere else (SPECIFY), 7) DON'T KNOW, 8) REFUSED | (a) Landfill (1, 2), (b) Litter/Sewer (3, 4, 5), (c) Container, (d) Other (-8), or (e) missing (-7); 6= “other specify” was recoded to existing categories. See supplemental tables for details. |
| Where respondent disposes of cigarette packs/cartons | What do you usually do with a cigarette pack or carton when it's empty? | Adult respondents who currently smoke manufactured cigarettes. | 1) Throw it away in an ash tray or cigarette disposal, 2) Throw it in the trash, 3) Throw it on the ground, 4) Recycle it, 5) Throw it out of a car window, 6) Somewhere else (SPECIFY), 7) DON'T KNOW, 8) REFUSED | (a) Landfill (1, 2), (b) Litter/Sewer (3, 5), (c) Recycle/reuse (4), (d) Fire/burn, or (e) Other (-8), or (f) missing (-7); 6= “other specify” was recoded to existing categories. See supplemental tables for details. |
| Where respondent disposes of empty roll-your own tobacco pouch | What do you usually do with a pouch of roll-your-own cigarette tobacco when it’s empty? | Adult respondents who currently smoke RYO cigarettes. | 1) Throw it away in an ash tray or cigarette disposal, 2) Throw it in the trash, 3) Throw it on the ground, 4) Recycle it, 5) Throw it out of a car window, 6) Somewhere else (SPECIFY), 7) DON’T KNOW, 8) REFUSED | (a) Landfill (1, 2), (b) Litter/Sewer (3, 5), (c) Recycle/reuse (4), (d) Other (-8), or (e) missing (-7); 6= “other specify” was recoded to existing categories. See supplemental tables for details. |
| Where respondent disposes of empty electronic nicotine product | What do you usually do with your disposable electronic nicotine product when it's empty? | Adult respondents who currently use ENP and use a disposable device type. | 1) Throw it away in the trash, 2) Throw it on the ground, 3) Recycle it, 4) Return it to a store or vape shop, 5) I have not gotten rid of an empty one, 6) Something else (SPECIFY), 7) DON'T KNOW, 8) REFUSED | (a) Landfill (1), (b) Litter (2), (c) Recycle/return/reuse (3, 4), (d) Have not gotten rid of an empty one (5), (e) Other (-8), or (f) missing (-7); 6= “other specify” was recoded to existing categories. See supplemental tables for details. |
| Where respondent disposes of empty pods or cartridges | What do you usually do with a pod or cartridge when it's empty? | Adult respondents who currently use ENP and use a prefilled pod or cartridge device type. | 1) Throw it away in the trash, 2) Throw it on the ground, 3) Reuse or refill it, 4) Recycle it, 5) Return it to a store or vape shop, 6) I have not gotten rid of an empty one, 7) Something else (SPECIFY), 8) DON'T KNOW, 9) REFUSED | (a) Landfill (1), (b) Litter (2), (c) Recycle/return/reuse (3, 4, 5), (d) Someone else' prod, (e) Have not gotten rid of an empty one (6), (f) Other (-8), or (g) missing (-7); 6= “other specify” was recoded to existing categories. See supplemental tables for details. |
| Where respondent disposes of e-product coils/atomizers | What do you usually do with the coils or atomizers for your electronic nicotine product after they no longer work? | Adult respondents who currently use ENP and use a refillable device type or a mod system device type. | 1) Throw them away in the trash, 2) Throw them on the ground, 3) Recycle them, 4) Return them to a store or vape shop, 5) I have not gotten rid of coils or atomizers, 6) Something else (SPECIFY), 7) DON'T KNOW, 8) REFUSED | (1) Landfill (1), (b) Litter (2), (c) Recycle/return (3, 4), (d) Have not gotten rid of coils or atomizers (5), (e) Other (-8), or (f) missing (-7); 6= “other specify” was recoded to existing categories. See supplemental tables for details. |
| Where respondent disposes of batteries after it no longer works | What do you usually do with the battery for your electronic nicotine product after it no longer works or is needed? | Adult respondents who currently use ENP and use a prefilled pod or cartridge device type, a refillable device type, or a mod system device type. | 1) Throw it away in the trash, 2) Throw it on the ground, 3) Recycle it, 4) Return it to a store or vape shop, 5) I have not gotten rid of an empty one, 6) I have not had a battery that no longer worked or was no longer needed, 7) Something else (SPECIFY), 8) DON'T KNOW, 9) REFUSED | (a) Landfill (1), (b) Litter (2), (c) Recycle/Return (3, 4), (d) Have not gotten rid of an empty one (5), (e) Other (-8), or (f) missing (6, -7); 6= “other specify” was recoded to existing categories. See supplemental tables for details. |
| Where respondent disposes of leftover/unused e-liquid | What do you usually do with leftover or unused e-liquid? | Adult respondents who currently use ENP and use a refillable device type. | 1) Throw it away in the trash, 2) Throw it on the ground, 3) Pour it into the sink or toilet, 4) Return it to a store or vape shop, 5) I have not gotten rid of leftover or unused e-liquid, 6) I have not had any leftover or unused e-liquid, 7) Something else (SPECIFY), 8) DON'T KNOW, 9) REFUSED | (a) Landfill (1), (b) Litter/Sewer (2, 3), (c) Recycle/Return/Reuse (4), (d) Have not gotten rid of leftover/unused e-liquid (5), (e) Gave it away, or (f) Other (-8), or (g) missing (6, -7); 6= “other specify” was recoded to existing categories. See supplemental tables for details. |
| Where respondent disposes of an empty bottle or container of e-liquid. | What do you usually do with a bottle or container of e-liquid when it's empty? | Adult respondents who currently use ENP and use a refillable device type. | 1) Throw it away in the trash, 2) Throw it on the ground, 3) Recycle it, 4) Return it to a store or vape shop, 5) I have not gotten rid of an empty one, 6) Something else (SPECIFY), 7) DON'T KNOW, 8) REFUSED | (a) Landfill (1), (b) Litter (2), (c) Recycle/return/reuse (3, 4), (d) Have not gotten rid of an empty one (5), or (e) Other (-8), or (f) missing (-7); 6= “other specify” was recoded to existing categories. See supplemental tables for details. |
